# Urinary RKIP/p-RKIP is a potential diagnostic and prognostic marker of clear cell renal cell carcinoma

**DOI:** 10.18632/oncotarget.16341

**Published:** 2017-03-18

**Authors:** Massimo Papale, Grazia Vocino, Giuseppe Lucarelli, Monica Rutigliano, Margherita Gigante, Maria Teresa Rocchetti, Francesco Pesce, Francesca Sanguedolce, Pantaleo Bufo, Michele Battaglia, Giovanni Stallone, Giuseppe Grandaliano, Giuseppe Carrieri, Loreto Gesualdo, Elena Ranieri

**Affiliations:** ^1^ Molecular Medicine Center, Section of Clinical Pathology, Department of Medical and Surgical Sciences, University of Foggia, Foggia, Italy; ^2^ Division of Urology, Department of Emergency and Organ Transplantation, University of Bari, Bari, Italy; ^3^ Division of Nephrology, Department of Emergency and Organ Transplantation, University of Bari, Bari, Italy; ^4^ Department of Pathology, University of Foggia, Foggia, Italy; ^5^ Division of Nephrology, Department of Medical and Surgical Sciences, University of Foggia, Foggia, Italy; ^6^ Division of Urology, Department of Medical and Surgical Sciences, University of Foggia, Foggia, Italy

**Keywords:** RKIP, phospho-RKIP, biomarkers, urine, clear cell renal cell carcinoma

## Abstract

Clear cell Renal Cell Carcinoma (ccRCC) causes over 13,000 deaths each year, and about 20,000 new cases/year in Europe. In most cases, the causes are unknown and, most importantly, there are no reliable biomarkers for the diagnosis and prognosis of this disease. The search for sensitive biomarkers for early diagnosis and prognosis of clear cell Renal Cell Carcinoma (ccRCC) is currently a fast growing field. We carried out proteomics analysis of 93 urinary samples of healthy subjects (HS) and patients affected by ccRCC, prostate cancer (PCa) and chronic kidney disease (CKD), that was able to successfully distinguish each group.

The most significant candidate biomarker was identified by mass spectrometry as Raf Kinase Inhibitor Protein (RKIP), a key regulator of cell signaling, already described in several cancer types as a metastasis suppressor. By combining ELISA, immunoblotting and tissue microarray, we demonstrated that, in ccRCC, urinary excretion of RKIP and its phosphorylated form (p-RKIP) reflected the tissue expression of these putative biomarkers. Baseline urinary RKIP, evaluated in an independent cohort of 56 ccRCC patients and 28 HS, successfully distinguished both groups and, most importantly, a cut-off value of 10 ng/mg/g Pr/uCr enabled a highly accurate prediction of Cancer-specific survival and Progression-free survival. Furthermore, p-RKIP was totally undetectable in both tissue and urine samples of ccRCC, showing a great potential for diagnostics purposes.

Our data indicate that urinary RKIP encompasses both the unphosphorylated and the phosphorylated form and that their combined evaluation can help in the diagnosis and prognosis of ccRCC.

## INTRODUCTION

Clear cell Renal cell carcinoma (ccRCC) has the third highest mortality rate among genitourinary cancers, with an estimated 13,000 deaths/year reflecting a steady trend toward an increased incidence of this cancer by 2–3% per year [1]. In Europe there are approximately 20,000 new cases each year and an annual death rate due to metastatic disease of 8,000 [1]. The pathogenesis of the ccRCC is still poorly understood, although some aspects of the lifestyle like cigarette smoking [2], obesity [3], hypertension [4], diabetes [5] and End Stage Renal Disease [6] have been recognized as common risk factors. The tumor arises from the renal epithelium; it is generally asymptomatic and, in approximately 30% of the patients, presents locally invasive or distal metastasis, at the time of diagnosis [7]. ccRCC is often accidentally diagnosed due to the extensive use of ultrasonography for other reasons, leading to an earlier diagnosis and probably better prognosis. However, the presence of metastatic disease in about one-third of the patients is correlated with less than 13 months survival [8, 9]. Thus, the key to making an early diagnosis for ccRCC is the identification of molecular tumor markers for testing in whole risk populations by easy routine assays. Many molecules such as C-reactive protein, CA15-3, αKlotho and some metabolic enzymes have been investigated, but none of these biomarkers have been definitively validated and their use is not recommended in clinical practice [10–14]. The development of omics approaches for high throughput screening of biological samples has improved the chances of finding reliable ccRCC biomarkers [15–19] even if to date, none of the identified biomarkers has been recognized as useful in clinical practice. Among biological fluids, urine is considered the gold standard for biomarker discovery studies on renal diseases probably due to the higher concentration of renal derived proteins [20]. Urinary proteomics by the so-called profiling technologies may overcome some of the limitations of laborious gel-based approaches, thus resulting more appropriate for clinical proteomics studies [21]. Recent reports demonstrated that urinary peptides and proteins could serve as specific biomarkers for chronic kidney diseases (CKD) [22–23] and uro-genital cancers [24]. However, most of the studies carried out so far have primarily compared patients to healthy controls, making it difficult to ascertain the specificity and sensitivity of the potential biomarkers. We designed a proteomics-based discovery approach that allowed the identification of Raf Kinase Inhibitor Protein (RKIP) and phosphor-RKIP (p-RKIP) as novel urine biomarkers of ccRCC, that can be useful for both differential diagnosis and prognosis of ccRCC.

## RESULTS

### SELDI protein profiling allows the identification of a ccRCC specific diagnostic model

**Figure 1:**
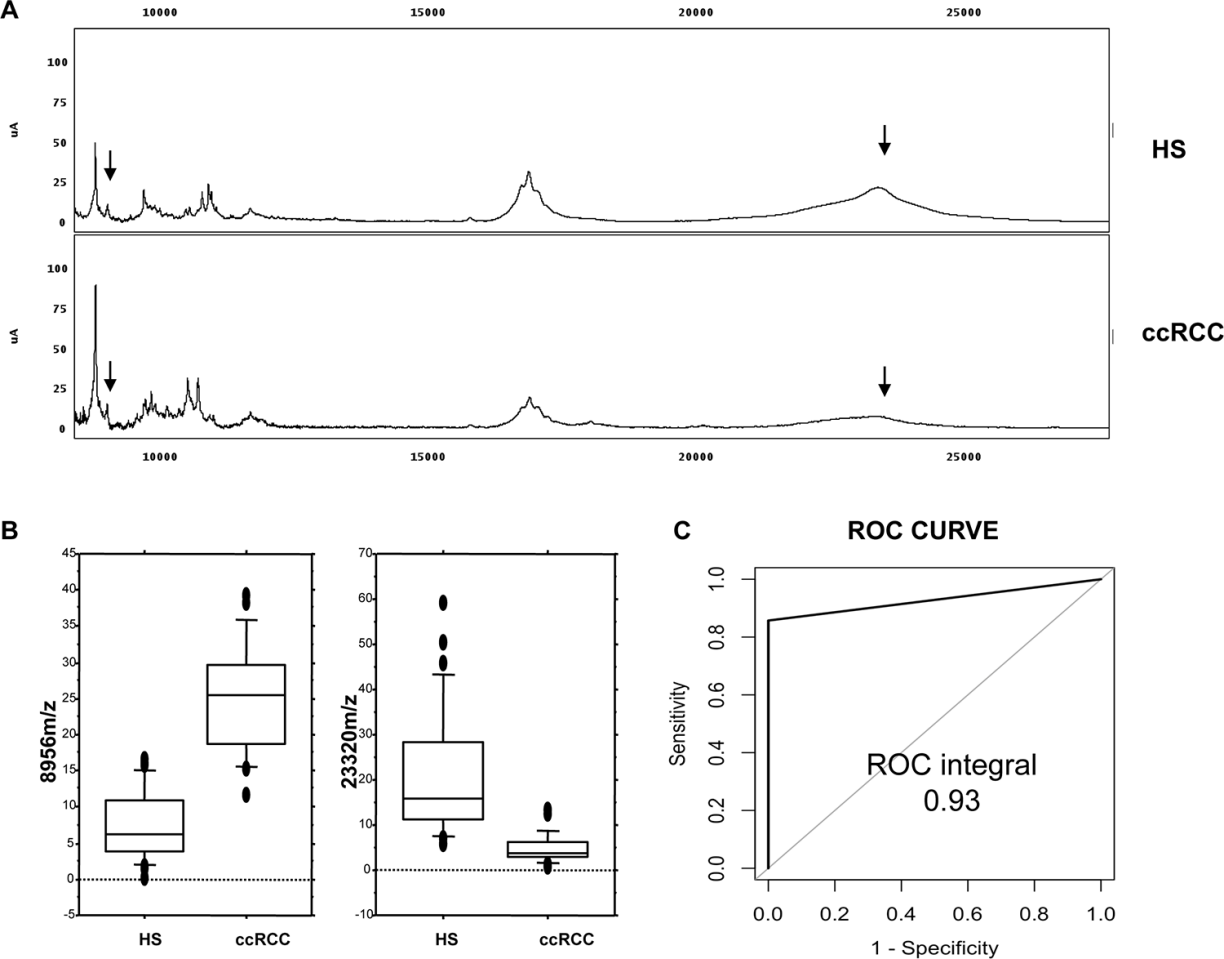
Recognition of ccRCC by urine protein profiling **(A)** Representative urine protein profiling of one ccRCC patient (ccRCC) and one Healthy Subject (HS); the arrows show the two mass peaks (8,956 m/z and 23,320 m/z, respectively) used for the CART analysis (Supplementary Table 1). Details of the classification and regression tree analysis reported in Supplementary Figure 1B. **(B)** Overall intensity of the 8,956 and 23,320 m/z in all the screened samples as represented by the box-plots. **(C)** Diagnostic power of the CART analysis applied to an independent testing set of ccRCC and HS urine samples.

To evaluate the presence of a specific urinary signature in ccRCC patients, we firstly compared urinary protein profiling in ccRCC and Healthy Subjects (HS). Within 101 mass peaks (clusters) shared between the groups, 68 were differently excreted (p-value < 0.05) in urine samples (Supplementary Table 1). Furthermore, the FDR, calculated according to Benjamini & Hochberg's method, showed 65 differently excreted mass peaks between the two groups. The proteomic dataset was managed by supervised statistical analysis (Classification and Regression Tree analysis- CART) in order to build an optimal classification tree serving to distinguish ccRCC patients. The most accurate classification tree evaluated the rate of excretion of two mass peaks (23,320 and 8,956 m/z) and was able to correctly classify 100% (14/14) HS and 87.5% (7/8) ccRCC patients in an independent testing set (Supplementary Figure 1), with an overall diagnostic power of 93% (Figure 1). The power analysis to detect differences between the two groups was 96% for the 8,956 peak and 94% for the 23,322 peak, at a level of significance of < 0.001. Of note, the classification tree was also able to distinguish ccRCC patients from prostate cancer (PCa) and chronic kidney disease (CKD) patients, with an accuracy of 70 % and 77%, respectively (Supplementary Figure 2).

### The 23,320 m/z SELDI predictor corresponds to raf kinase inhibitor protein

**Figure 2:**
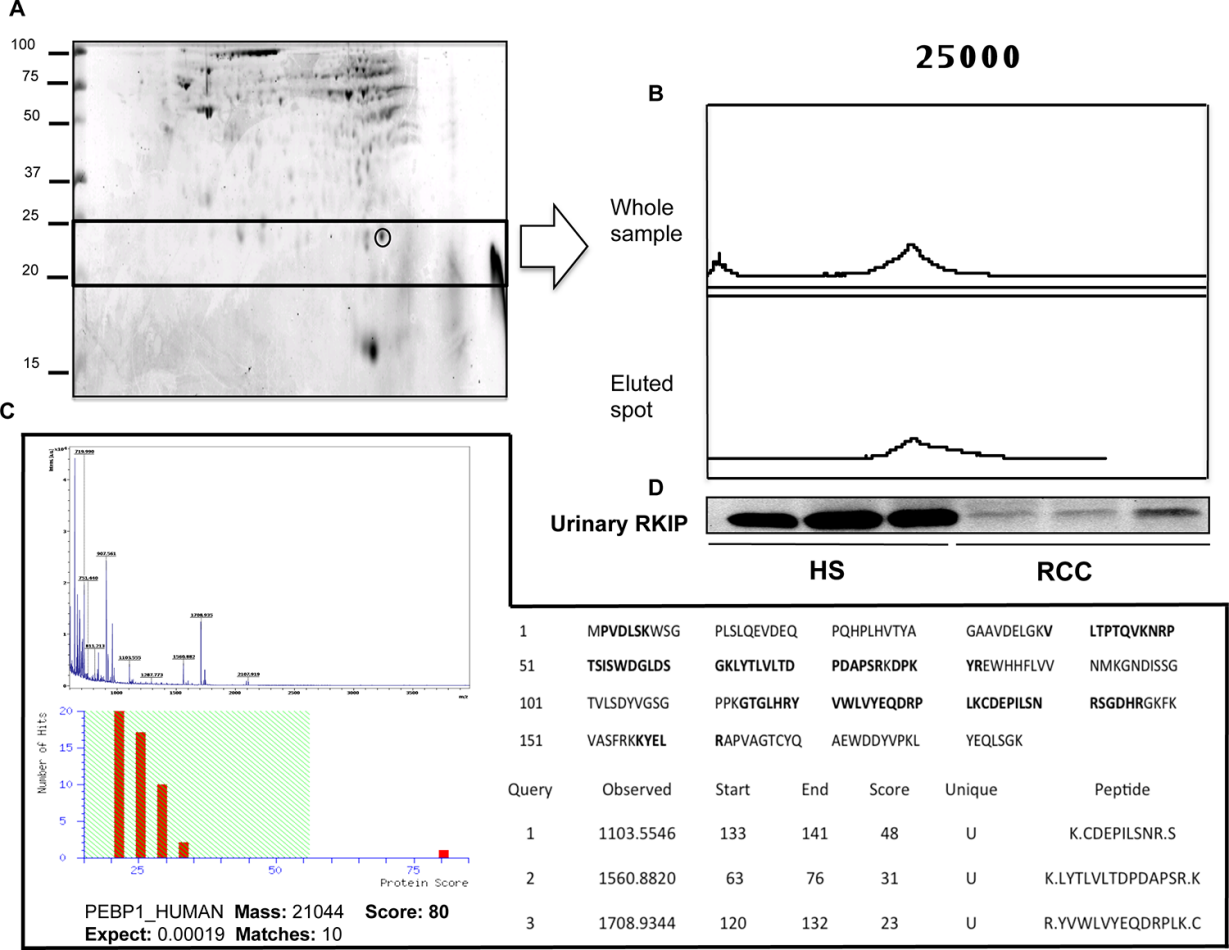
Purification and identification of RKIP **(A)** Two-dimensional gel map of a healthy subject. Gel spot corresponding to the 23,320 m/z peak is highlighted. **(B)** SELDI-TOF/MS profile of the whole sample and of the spot eluted from the gel; **(C)** MALDI TOF/MS-MS sequencing of the eluted spot: MALDI spectra and mascot score after Peptide Mass Fingerprinting are provided in the left part of the panel; Protein sequence with matching peptides (in bold) and the sequence of unique peptides after MS/MS analysis are shown in the right part of the panel. **(D)** Validation of urinary RKIP differential excretion between ccRCC and HS as obtained by immunoblotting. PEBP_1 is an alternative name for Raf Kinase Inhibitor Protein (RKIP). SELDI profiling of all the gel spots eluted from the gel is reported in Supplementary Figure 3

In order to ascertain the identity of the 23,320 m/z mass peak, we carried out a two-dimensional electrophoresis experiment (Figure 2A) followed by passive elution of the protein spots in the mass range between 20 and 25 kDa and SELDI analysis to identify the one corresponding to the <23,320 m/z peak (Supplementary Figure 3 and Figure 2B) in the urine SELDI profiling. After recognizing the protein spot matching the SELDI mass peak, MALDI-TOF/MS-MS analysis allowed this candidate biomarker to be identified as Raf Kinase Inhibitor Protein (Figure 2C). The reduced urinary excretion of RKIP in ccRCC Vs. HS was confirmed by immunoblotting (Figure 2D).

### RKIP urinary excretion as diagnostic marker of ccRCC

**Figure 3:**
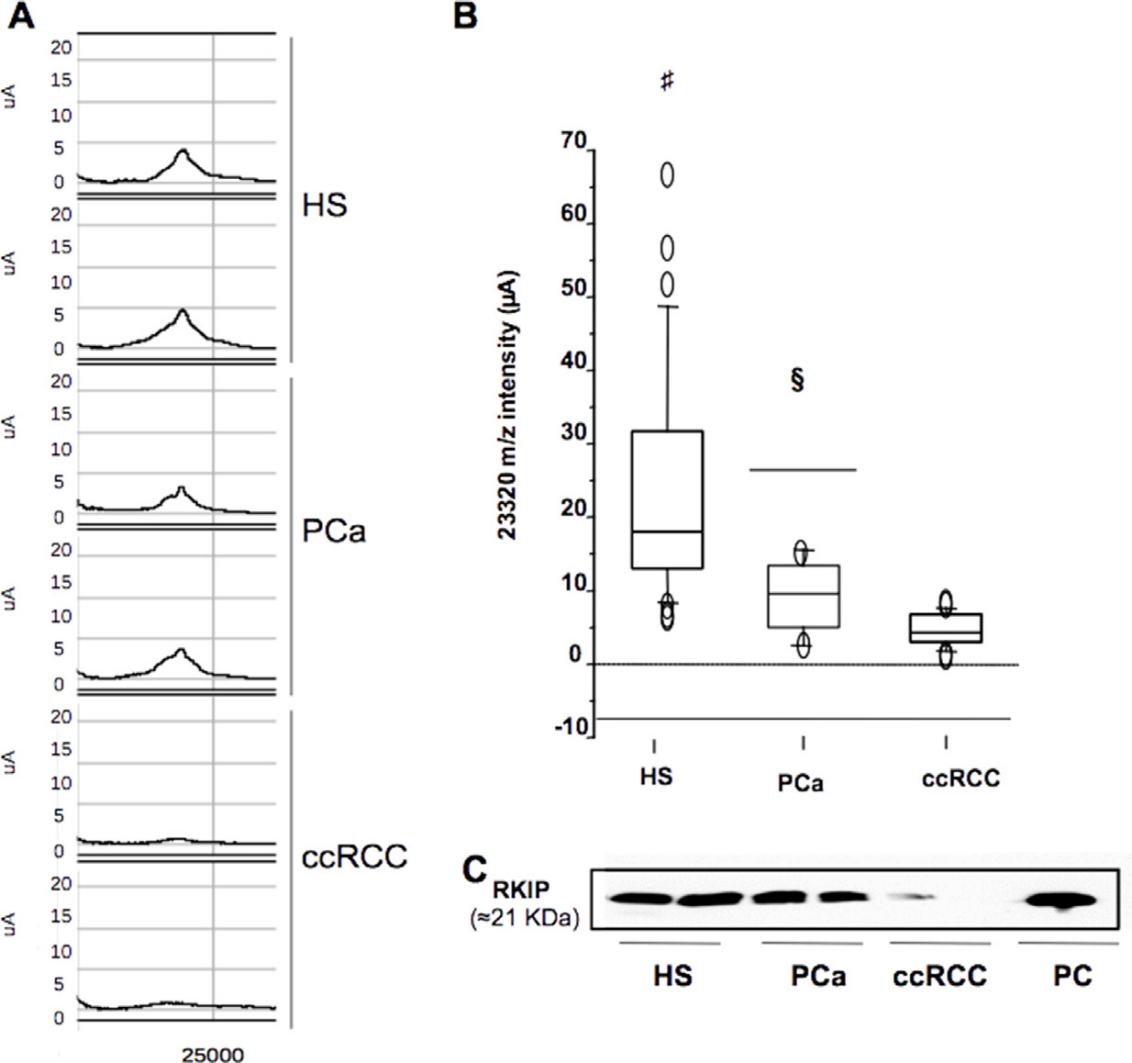
Selective reduction of urinary RKIP in ccRCC **(A)** Urinary expression of ~23,322 m/z mass peak (RKIP) in ccRCC vs. PCa and HS samples as measured by SELDI analysis. **(B)** Overall intensity (μA) of RKIP (23,320 m/z) in all samples as represented by the box-plots. **(C)** urinary levels of RKIP in ccRCC Vs. PCa and HS as measured by western blotting. Each sample used in WB analysis was a pool of 4 samples. HS= Healthy Subjects; PCa= Prostate cancer; ccRCC= clear cells Renal Cell Carcinoma; PC: positive control (protein extract from Rat hyppocampus); #: HS vs RCC p-value < 0.001; §:PCa vs ccRCC p-value < 0.01 (Mann Whitney-U test).

**Figure 4:**
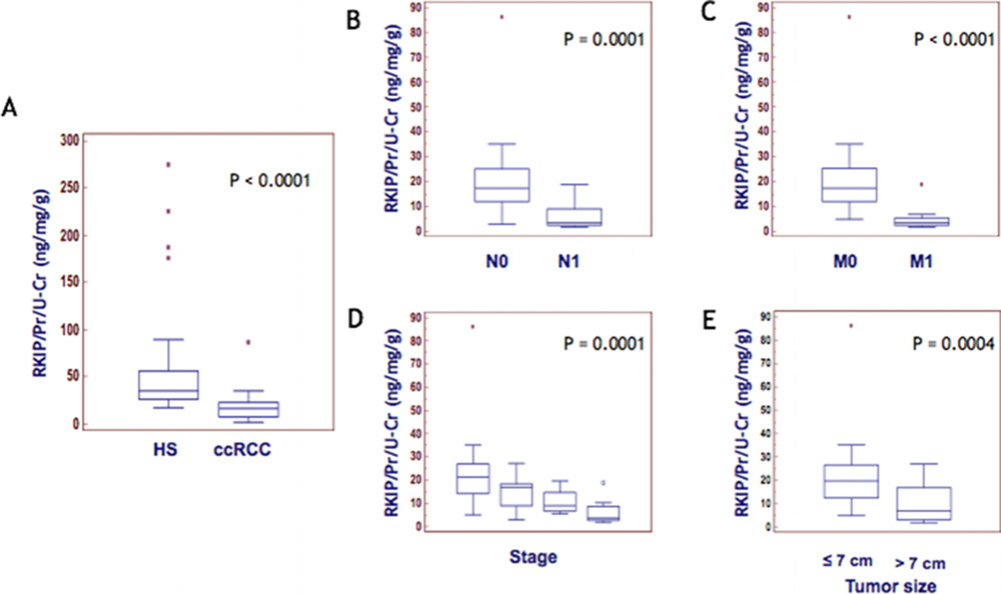
RKIP correlation with disease progression Median levels of urinary RKIP in HS Vs. ccRCC **(A)** and in ccRCC patients stratified according to lymph node involvement **(B)** visceral metastases **(C)**, clinical stage **(D)** and tumor size **(E)**.

In order to validate the significantly differential urine excretion of RKIP between ccRCC and PCa patients observed by SELDI profiling (Figure 3A and 3B), we carried out immunoblotting analysis on urine samples of 8 patients from each group. As expected, RKIP urinary excretion appeared significantly reduced in ccRCC patients vs. HS and PCa (Figure 3C). We further tested, by ELISA, RKIP urinary excretion in an independent cohort of 56 ccRCC patients and 28 HS. The ccRCC group encompassed patients with different tumor grade, stage and size, and median follow-up was 41 months. The analyses were carried out blinded in order to validate the utility of urinary RKIP as an early diagnostic marker and to evaluate its prognostic value. Median RKIP urinary levels were significantly lower in ccRCC patients than HS (35.05 vs 16.44; Figure 4A). RKIP values were significantly correlated with lymph node involvement (Figure 4B), presence of visceral metastases (Figure 4C), clinical stage (P=0.0001; Spearman correlation: rs=-0.63, P<0.0001) (Figure 4D) and tumor size (≤ 7 vs > 7 cm; P = 0.0004) (Figure 4E). No correlation was found between RKIP levels and Fuhrman grade (P=0.2) (data not shown).

### RKIP urinary excretion as prognostic marker of ccRCC

#### Cancer-specific survival (CSS)

To evaluate the association between patients’ survival and the urinary levels of RKIP, we classified the entire population by high versus low RKIP expression levels according to the cut-off provided by ROC curve analysis. A cut-off of 10 ng/mg/g Pr/uCr provided the optimal balance between sensitivity and specificity for both cancer-specific survival and progression-free survival (Supplementary Table 2).

**Figure 5:**
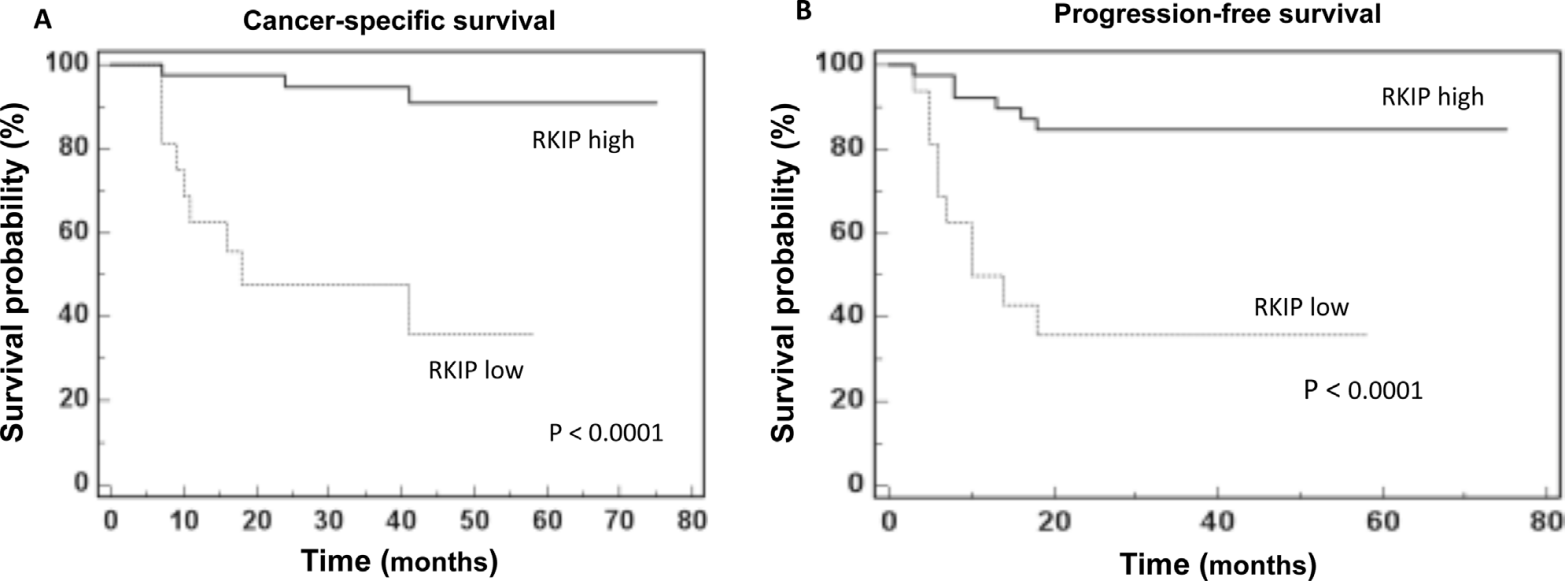
RKIP prognostic value Kaplan-Meier survival curves for **(A)** Cancer-specific survival (CSS) and **(B)** Progression free survival (PFS) as calculated by an optimal cut-off value of 10 ng/mg/g Pr/UCr.

After a median follow-up of 41 months (95% CI: 29.9 – 45.0), 12 patients had died of ccRCC. Kaplan-Meier survival curves for CSS, stratified by RKIP urinary levels, are shown in Figure 5A. CSS was significantly decreased in patients with low levels of RKIP (P<0.0001). Univariate analyses of the predefined variables showed that age, tumor size, pathological stage, presence of visceral metastases, TNM stage, Fuhrman grade, and low urinary levels of RKIP were significantly associated with the risk of death (Supplementary Table 3). At multivariate analysis by Cox regression modeling, tumor size, the presence of visceral metastases, high Fuhrman grade and low urinary levels of RKIP were independent adverse prognostic factors for CSS (Supplementary Table 3).

#### Progression-free survival (PFS)

After surgery, 16 patients showed disease progression, after a median PFS of 39 months (95% CI: 28.7 – 45.0). Kaplan-Meier survival curves for PFS, stratified by RKIP urinary levels, are shown in Figure 5B. The PFS was significantly decreased for patients with low levels of RKIP (P<0.0001). Univariate analyses for the predefined variables showed that tumor size, pathological stage, presence of visceral metastases, TNM stage, Fuhrman grade, and low levels of RKIP were significantly associated with the risk of progression (all P<0.0001). At multivariate analysis, only tumor size, presence of visceral metastases and low levels of RKIP were independent adverse prognostic factors for PFS (Supplementary Table 4).

### Dimeric urinary p-RKIP distinguishes ccRCC from chronic kidney disease (CKD)

**Figure 6:**
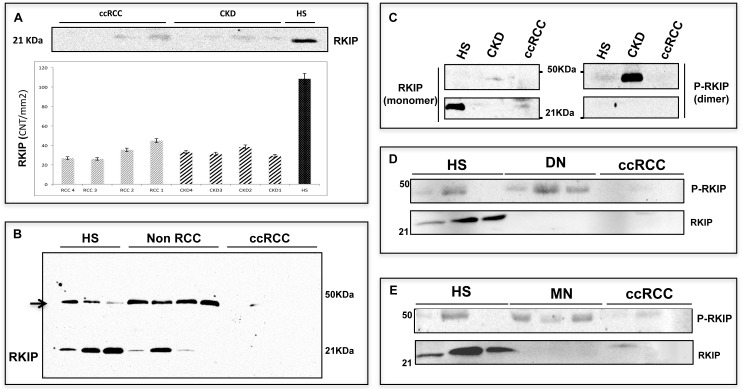
p-RKIP diagnostic value **(A)** Urinary excretion of RKIP (~21 KDA) as measured by immunoblotting in ccRCC and CKD patients and its densitometric quatitation (CNT per mm2) in each patient. **(B)** Immunoblotting analysis of RKIP in HS, non RCC (prostatic cancer) and ccRCC urine samples: other then the putative RKIP (~21 KDa), a second band of approximately 50 KDa (indicated by the arrow) appears in all samples with the exception of ccRCC. **(C-E)** Urinary excretion of RKIP and p-RKIP in 1 ccRCC vs 1 CKD patient (C) and validation in ccRCC Vs. DN **(D)** and Vs. MN **(E)**. CKD = Chronic Kidney Disease; DN= Diabetic Nephropathy; MN= Membranous Nephropathy. Full-size immunoblotting are reported in Supplementary Figure 4.

**Figure 7:**
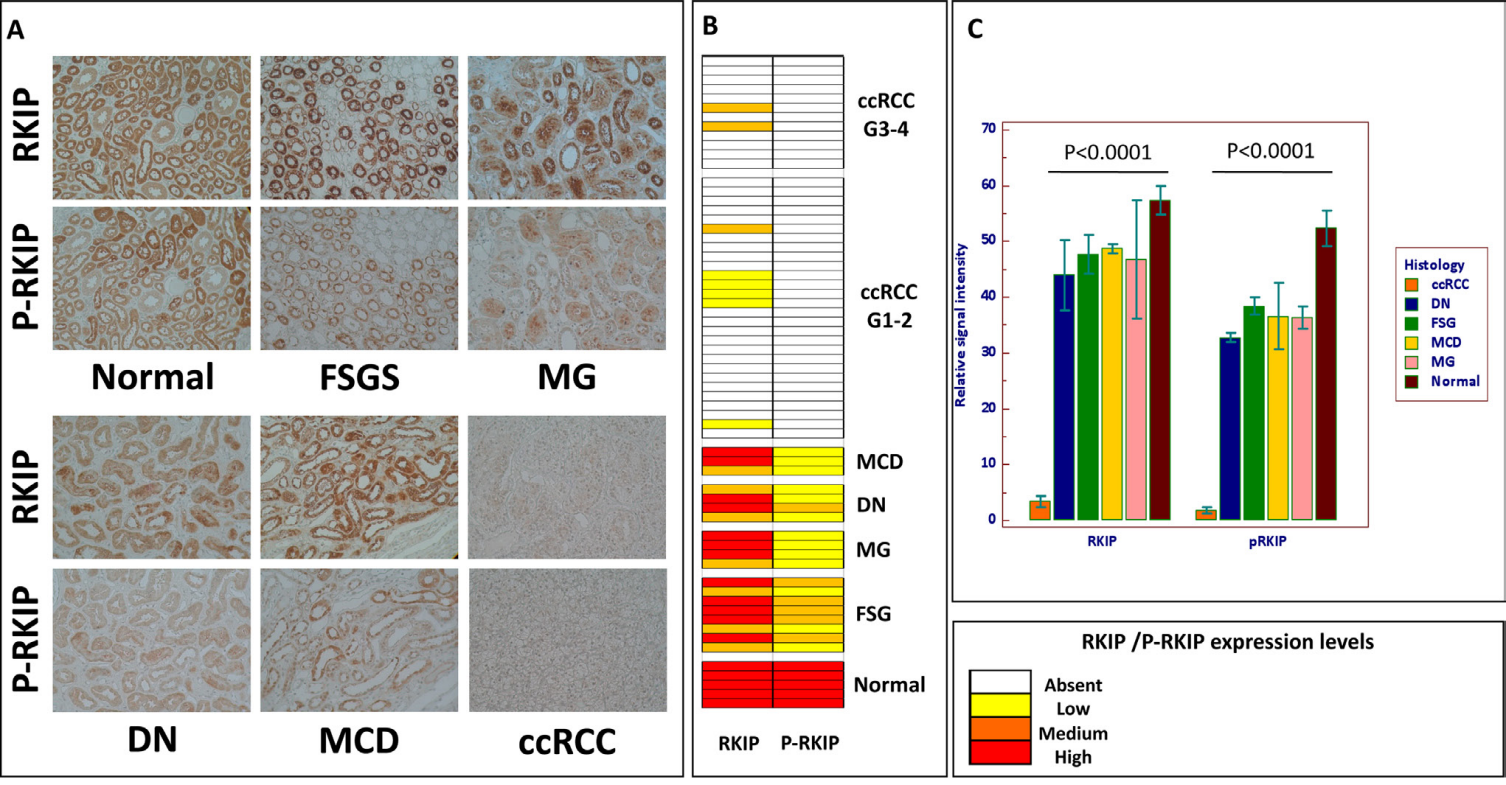
RKIP and p-RKIP tissue expression Immunohistochemical staining of RKIP and p-RKIP proteins in tissue microarrays of normal human kidney (n. 5), chronic kidney diseases (CKD n. 19), and clear cells Renal Cell Carcinoma (ccRCC n. 40) specimens **(A)**. Heat map summarizing RKIP and p-RKIP staining in 64 patients **(B)**. RKIP and p-RKIP tissue expression quantization **(C)**. Original magnifications 20×. CKD group included 8 Focal Segmental Glomerulosclerosis (FGS); 4 Membranous Nephropathy (MG); 4 Diabetic Nephropathy (DN); 3 Minimal Change Disease (MCD).

Immunoblotting analysis of RKIP in the urine samples of ccRCC and CKD patients revealed no significant difference in the putative RKIP band of ≈ 21 kDa (Figure 6A). Of note, a second band at approximately 50 kDa often appeared in our experiments and seemed to be correlated with the diagnosis (Figure 6B). Since Deiss and co-workers [25] have already described a dimeric phosphorylated form of RKIP at about 50 kDa, we evaluated the hypothesis that the accessory band might represent this form. Immunoprecipitation by RKIP of the urine sample of one HS, one CKD and one ccRCC patient was done, then blotting by RKIP or by p-RKIP monoclonal antibody to phosphoserine 153 (pS153), required for RKIP dimerization. As expected, RKIP blotting showed comparable low signals in both ccRCC and CKD but, in the latter sample, p-RKIP blotting revealed a second signal at about 50 kDa (Figure 6C). These data suggested that RKIP may be excreted in urine samples in both monomeric and dimeric form and confirmed that protein dimerization is mediated by S153 phosphorylation. We further analyzed the urinary excretion of RKIP/p-RKIP in 6 CKD patients, 3 with biopsy-proven diabetic nephropathy (Kimmestiel-Wilson glomerulosclerosis) and 3 with biopsy-proven non diabetic glomerulopathy (Membranous Nephropathy) (Figure 6D–6E). RKIP blotting showed indistinguishable low levels of the monomeric RKIP in both ccRCC and CKD patients compared to HS, while p-RKIP blotting allowed a clear differentiation of the CKD from the ccRCC group (Figure 6D–6E). To evaluate whether the urinary excretion of RKIP and p-RKIP may reflect kidney tissue expression, we carried out TMA analysis on 5 apparently normal kidney sections compared to 40 ccRCC and 19 CKD. RKIP was detectable in all normal tissue (H-score = 3 for all) and CKD patients (H-score 3 or 2 for all) but only slightly expressed in 8/40 ccRCC tissue (H-score 2 or 1). Interestingly, the phosphorylated form, p-RKIP, was highly expressed in all normal kidney tissues (H-score= 3), reduced in CKD (H-score = 1-2) and totally undetectable in all ccRCC tissue specimens, independently of tumor grade, presence of visceral metastases, lymph node involvement and tumor size (P<0.0001) (Figure 7).

## DISCUSSION

The lack of molecular biomarkers for the early detection and classification of ccRCC is one of the major obstacles to improving the clinical outcome of ccRCC. Here, we report the utility of urinary detection of RKIP and p-RKIP to improve the diagnosis and determine the prognosis of ccRCC. Urine protein profiling of ccRCC patients and matched healthy subjects allowed a number of significant differently excreted urine mass peaks to be identified in these two groups.

Furthermore, the combined evaluation of these two predictors allowed the correct classification of ccRCC and HS, with 93% accuracy. Interestingly, the classification model was able to successfully identify ccRCC compared to PCa and CKD patients (Supplementary Figure 2). However, we handled these findings with care since it is well known that even small variations in the training data can lead to big changes in the tree generated by a CART analysis. Thus the stability of the diagnostic pattern may be confirmed only after multiple validations on independent testing sets. Furthermore, CART uses only one feature each time, not considering possible interactions between variables (multicollinearity) and combinations of variables for their classification capability. For these reasons, we limited the use of this approach to the preliminary and rapid selection of a possible set of features to be further sequenced and validated. The mass peak of 23,322 m/z was the most important variable in all the CART analyses (Figure 1, Supplementary Figure 1 and 2) so we then focused on its identification and validation. It was purified by combining 2-DE separation and SELDI-TOF/MS profiling for peak confirmation and MALDI-TOF/MS-MS for sequencing (Figure 2). The gel spots in the mass range between 20 and 25 kDa were purified from the 2-DE map and run on SELDI ProteinChip to pinpoint the one corresponding to the 23,322 m/z peak, that was finally identified as RKIP by MALDI-TOF/MS-MS. Of note, the signal obtained by SELDI profiling was slightly different from the predicted molecular weight of RKIP (<23 kDa Vs. < 21 kDa) as a consequence of the lower accuracy of SELDI for proteins of medium and high molecular weight range. Interestingly, a slight signal at 23,320 m/z was also produced by some other protein spots (Supplementary Figure 3), even if MALDI sequencing of these proteins did not produce any results and it was not possible to verify whether they were different forms of RKIP or other proteins sharing the same molecular weight. However, western blotting analysis confirmed the trend toward a significant reduction of urinary RKIP previously observed in the SELDI experiments and the potential utility of this biomarker to distinguish ccRCC patients from HS and prostatic cancer (Figure 3). Urinary RKIP showed a diagnostic and, most importantly, prognostic value, as demonstrated in an independent cohort of 56 ccRCC patients. Urinary RKIP was, in fact, significantly less excreted in ccRCC patients than in healthy subjects and was also correlated with lymph node involvement, staging, presence of metastases and tumor size at diagnosis (Figure 4). Furthermore, Kaplan-Mayer curves revealed that ccRCC patients with less than 10 ng/mg/g Pr/uCR at diagnosis had a higher risk of disease progression and death after a median of 41 months follow-up (Figure 5). Urinary RKIP did not allow ccRCC to be distinguished from patients with chronic kidney disease (Figure 6A) but our experiments demonstrated that RKIP may be released in urine samples both as a free monomer and a phosphorylated dimer (p-RKIP) and that the evaluation of the latter form may be useful to distinguish ccRCC from CKD patients (Figure 6D–6E). To confirm this point, we analysed RKIP e p-RKIP expression in tissue samples of 40 ccRCC, 19 CKD and 5 normal kidney tissues by Tissue Micro Array (TMA) analysis. As expected, RKIP was significantly reduced, almost absent, in ccRCC compared to CKD and normal kidney while p-RKIP was expressed in HS and CKD but completely undetectable in all ccRCC tissue (Figure 7). Our data about RKIP tissue expression are in line with those recently published by Hill et al. [26]. In short, we describe, for the first time, the absence of p-RKIP in ccRCC in both tissue and urine samples of ccRCC patients.

It is worth noting that RKIP/p-RKIP may be involved in the pathophysiology of both ccRCC and CKD by triggering the Epithelial Mesenchymal Transition (EMT), [27–28] a key process mediating progression and fibrosis in ccRCC and CKD. We speculated that impaired expression of RKIP might activate the EMT both in CKD and ccRCC, albeit through distinct mechanisms. It has been reported that in ccRCC, the EMT is correlated with an increased expression of SNAIL-1, a well-known suppressor of RKIP [29], leading to an increased activation of MAPK-ERK signaling [30–31]. On the contrary, RKIP expression in tissue of CKD patients was not significantly different from in normal kidney tissues and interestingly, p-RKIP was also detectable. As reported by Deiss and co-workers [25], RKIP dimer formation is necessary to switch its target from RAF-1 to G protein-coupled receptor kinase (GRK) 2 and the phosphorylation of Ser-153 also increases the affinity of RKIP for GRK2. GRK2 is a ubiquitous member of the G protein-coupled receptor kinase (GRK) family that plays a central, integrative role in signal transduction cascades [32]. PI3K, one of the targets of GRK2 [32], has been associated to the EMT and fibrosis in CKD patients so we hypothesize that dimeric RKIP, observed in urine of CKD patients, may reflect GRK2-mediated activation of PI3K signaling and the EMT in these patients. In this scenario, the reduced RKIP observed in urine samples of both ccRCC and CKD patients may depend, in ccRCC, on SNAIL-1 mediated down-regulation, and in CKD, on phosphorylation-mediated RKIP dimerization. Overall, our data indicate that urinary evaluation of RKIP and p-RKIP may permit ccRCC to be distinguished from other urological malignancies and CKD, and provide an explanation of the pathophysiological significance of the disappearance of both the monomeric and the dimeric form of this protein in urine samples of ccRCC patients.

The present study has some limitations. Firstly, our results need to be validated in large multicentric studies; further, other urological malignancies, such as bladder cancer, should be investigated in order to complete the evaluation of urinary RKIP in urological diseases. The pathophysiological mechanism we have hypothesized needs to be confirmed by mechanistic experiments in animal models. Despite these limitations, the results of this pilot study contribute to improve our knowledge of the pathophysiology of ccRCC and provide scientific evidence of the utility of this new biomarker to make a better prognosis and diagnosis of the disease.

## MATERIALS AND METHODS

### Study design

The aim of the study was to identify and validate noninvasive biomarkers of clear cell Renal Cell Carcinoma (ccRCC). We carried out urine protein profiling analysis of affected patients and matched controls to recognize a proteome-based pattern serving to differentially classify each group. The initial discovery and validation cohorts included 93 patients with early stage ccRCC, prostate cancer (PCa), chronic kidney disease (CKD) and healthy subjects (HS). Multivariate analysis of the protein pattern was used to recognize the combination of protein mass peaks that could classify the groups with the highest sensitivity and specificity. The most important predictor in this pattern was purified, identified and validated by immunoblotting and by ELISA. Tissue Micro Array (TMA) analysis was used to demonstrate that the biomarker's urine excretion reflected its tissue expression. A second cohort of 56 ccRCC patients and 28 healthy controls was used as an independent validation set in order to define the diagnostic and prognostic value of the biomarker. Baseline urine evaluation of RKIP in this cohort was correlated to the grade, the stage and, most importantly, to cancer-free survival and progression-free survival. To improve the diagnostic and prognostic power of RKIP, the analyses were extended also to (ser153) phosphorylated RKIP in order to correlate its tissue and urinary levels.

### Patients

In the initial discovery phase, 93 patients were enrolled. They included 22 ccRCC, 13 prostate cancer (PCa), 22 chronic kidney disease (CKD) and 36 healthy subjects (HS), enrolled from 2007 to 2014 by the Nephrology Units at the Universities of Foggia and Bari and by the Urology Unit at the University of Bari. Only ccRCC patients showing no distal metastasis or lymph node infiltration, at histopathological examination, and healthy volunteers with no evidence of renal function abnormalities, cancer and/or other systemic diseases were enrolled in this phase of the study. Other control groups were Prostatic cancer patients and patients affected by Chronic Kidney Disease. Detailed clinical characteristics of each group are shown in Supplementary Table 5. In addition, urinary RKIP was preoperatively measured in an independent cohort of 56 ccRCC patients who showed different tumor size, pathological stage, presence of visceral metastases, and Fuhrman grade and in 28 healthy adult volunteers with no evidence of malignancy. All patients were classified according to age, family history, stage and tumor grade, comorbidities (such as hypertension, diabetes and nephropathy) and drugs taken, and checked for pathogens (HCV, HBV, HIV). Written informed consent to take part was given by all participants. All patients were preoperatively staged by thoraco-abdominal Computed Tomography (CT) or Magnetic Resonance Imaging. Tumor staging was reassigned according to the 7th edition of the AJCC-UICC TNM classification. The 2004 World Health Organization and Fuhrman classifications were used to attribute histological type and nuclear grade, respectively. The study was approved by the local ethics committee (Study N. 4239 prot. N. 972/CE) and was carried out in accordance with the International Ethical Guidelines and Declaration of Helsinki. Clinical characteristics of the patients enrolled in the validation set are shown in Supplementary Table 6.

### Sample collection management and analysis by SELDI-TOF/MS

Second-voided urine samples were collected from each patient and, for ccRCC patients, before nephrectomy. The samples were processed as previously reported [20] and stored at -80°C until use. Prior to each analysis, the instrument performance was checked by the ProteinChip OQ kit (BIORAD). All the samples were analysed within a short time frame when the machine was working to standard. The SELDI analysis was carried out by loading 10 μg proteins from each patient in duplicate on CM10 ProteinChip array (BIORAD, cat. C57-30075) and following our previously described protocol [23]. After acquisition, the spectra were analysed by Protein chip DataManager™ 3.5 software (BIORAD, Hercules, CA, USA). The analysis was performed in a m/z range from 3,000 to 30,000 daltons, considering as real peaks those with S/N and a valley depth ratio greater than 4. All the spectra were normalized by means of total ion current. The reproducibility of the SELDI analysis, assessed as previously described [20], was comparable to that in our previous works [22, 23]. Only mass peaks showing a statistically significant different expression (p<0.05) between cases and controls were considered for further analyses.

### Univariate and multivariate statistical analysis of SELDI dataset

DataManager™ software (BIORAD) was used to identify a list of shared mass peaks (clusters) among all sample groups, then Mann-Whitney non parametric test was used to find differently excreted urine peaks (p-value < 0.05) between the ccRCC patients and HS. For multivariate analysis, all the samples (22 ccRCC and 36 HS) were initially considered. The intensities (μA) of all the mass peaks were transferred to Biomarker Pattern Software (BPS^®^ -BIORAD) to build-up the optimal classification tree able to distinguish the samples from each group with the least error. We set 4 as the maximum number of splits in the classification tree in order to limit overfitting issues. The best classification tree was then scored by 10 times internal cross-validation. Furthermore, the samples were randomly divided three times into an independent training set (22 HS Vs. 14 ccRCC) and a testing set (14 Hs Vs. 8 ccRCC) and run to construct and validate the three classification trees (Supplementary Figure 1). The independent testing sets were then scored using the optimal classification tree, to evaluate the classification power on blinded data sets. The performances of the classification tree after internal cross-validation and three independent testings were finally compared in order to verify the reproducibility of the results. Sensitivity was defined as the probability of predicting ccRCC, while specificity was defined as the probability of predicting HS or other control groups.

### Two-dimensional electrophoresis (2-DE) and MALDI-TOF/MS-MS analysis

To identify the 23,320 m/z peak, urine proteins from one healthy subject were separated by 2D-PAGE as previously reported [33]. Gels were stained by Colloidal Coomassie Blue G-250 and scanned with a flat-bed ImageScanner (Amersham Pharmacia Biotech) to generate digitized images. All protein spots (gel pieces) on 2-DE gels included in the 20-25 kDa mass range were manually excised and washed with H2O for 2 h at RT. Each piece was divided into two parts to allow passive elution and in-gel tryptic digestion at occurrence. For passive elution, proteins were allowed to diffuse out of the gel overnight at 37 °C by incubation in 30 μl of 0.1M sodium acetate, 0.1% SDS, pH 8.2. Then, the supernatant containing the eluted proteins of each gel spot was analyzed by SELDI-TOF/MS and compared to the protein profiling of the whole sample to find the proteins with 23,320 m/z. The protein spot corresponding to the candidate biomarker of 23,320 m/z underwent in-gel tryptic digestion as described by Shevchenko, A. et al [34] then the peptide mixture was spotted on the MALDI target plate for analysis. The MALDI mass spectra were acquired on an Autoflex III™ TOF/TOF 200 instrument with smartbeam™ laser technology. All spectra were acquired in reflecting mode with 200 Hz laser frequency, a delayed extraction time of 10, in the 500-3500m/z range. LIFT™ MS-MS spectra were externally calibrated using abundant fragment ion peaks derived from bradykinin [1–7], angiotensin I, angiotensin II, substance P, bombesin, ACTH 1-17, and ACTH 18-39, ACTH1_24, Insulin_B. Precursor ions for MS-MS analysis were selected with a timed ion selector at a resolution of approximately 450. FlexAnalysis 3.3 software was used for spectra processing then Biotools 3.2 and MASCOT search algorithm (http://www.matrixscience.com/) online version 2.4 against the NCBInr and Swissprot databases were used for protein identification using the following parameters: Homo Sapiens as taxonomic category, trypsin as enzyme, carbamidomethyl as fixed modification for cysteine residues, oxidation of methionine as variable modification, and one missing cleavage and 50 ppm as mass tolerance for the monoisotopic peptide masses and 0,3 Da mass tolerance for MS-MS analysis. The minimal probabilistic MASCOT score value needed for a p<0.05 significant identification was 56 for Swissprot and 67 in NCBInr.

### Immunoblotting and IP

Four ml urine samples from each patient were concentrated by 3kDa cut-off AMICON ULTRA filter devices (Millipore, Billerica, MA, USA), then the protein content was determined by Bradford assay and 80 μg urine proteins were diluted in DB3 buffer (UREA 9M, CHAPS 2% and DTT 100 mM) up to 300 μl and newly concentrated by AMICON ULTRA filter devices to obtain the buffer exchange. The protein samples were separated by 12% SDS-PAGE in denaturing and reducing conditions then, after NC membrane blotting, immunoblotting was carried out using specific antibodies for RKIP (Abcam Cambridge, UK cat. Ab76582) and p-RKIP (Santa Cruz Biotechnology cat. sc-32623). HRP-Goat anti Rabbit (Santa Cruz Biotechnology cat. Sc-2030) and HRP-Goat anti mouse (Santa Cruz Biotechnology cat. Sc-2031) were used as secondary antibodies for anti-RKIP and anti-p-RKIP, respectively. Clarity ™ Western ECL substrate (BIORAD, Hercules, CA, USA) was used to detect RKIP and p-RIKP through the Versadoc Molecular Imager™ (Bio-Rad). Results of densitometry analysis were expressed in arbitrary units. Data were normalized to the total protein content in each sample. Immunoprecipitation (IP) of RKIP was carried out by Protein G-coupled Dynabeads (Life Technologies, USA). Briefly, 50 μg RKIP antibody diluted in fresh made PBS were conjugated to 1.5 mg dynabeads, then cross-linked to the beads by bis(sulfosuccinimidyl)suberate (BS_3_). Two hundred μg total urine proteins from each sample were added to the antibody-beads complexes then IP was carried out according to the manufacturer's instructions. At the end of the procedure, the eluted RKIP was diluted in DB3 buffer and analysed by SDS-PAGE in duplicate. One duplicate underwent immunoblotting by RKIP and one by p-RKIP antibody.

### RKIP ELISA assay

The concentration of urinary RKIP was measured by a commercial ELISA kit (MyBioSource, San Diego, USA), according to the manufacturer's instructions, adapted to the urine sample. Briefly, 100 μl of urine and 100 μl reconstituted standard were loaded, in duplicate, and incubated at 37°C for 2 hours. After incubation, the wells were washed 3 times in washing solution and subsequently incubated for 1 hour at RT with the Detection Reagent A (tracer detection antibody specific for the immunocomplex RKIP/RKIPAb, conjugated with a biotin molecule) and after washing, the Detection Reagent B (HRP-conjugated avidin) was added to each well and incubated for 30 minutes at RT. Then, the antigen-antibody complex was revealed by adding the chromogenic TMB substrate and reading at 450nm OD. Urinary RKIP in each sample was normalized to the ratio of proteinuria/creatinuria (Pr/uCr) and RKIP concentration was expressed as ng/mg/g Pr/uCr.

### Immunohistochemistry and tissue microarray construction

Six high-density tissue microarrays (TMA) were used for RKIP and pRKIP immunostaining. Archived formalin-fixed paraffin-embedded renal tissue samples for a total of 64 cases were obtained (5 samples of normal renal tissue, 19 samples derived from patients with chronic kidney disease, and 40 samples of ccRCC). All tumor cores were identified by two uro-pathologists. These were selected by identifying representative tumor-containing slides and were used to assign the original tumor grade in each case. Three-mm cores were removed from the selected area (region of interest) using a needle punch. These 3- mm donor cores were subsequently embedded in previously arranged recipient paraffin blocks through a precisely spaced 15-hole array pattern. Core positions in the recipient paraffin block were noted on a TMA map. After paraffin cooling, the recipient blocks were cut in the microtome and used for immunohistochemistry. Immunohistochemical evaluation of RKIP and pRKIP protein expression was carried out on paraffin-embedded tissue sections. TMA were deparaffinized and rehydrated through xylene and graded alcohol series. Slides were subjected to specific epitope unmasking by microwave treatment (700W) in citrate buffer (0.01M pH=6.0). After antigen retrieval, TMA were incubated for 10 min with 3% H2O2 to block endogenous peroxidase activity. Sections were treated with serum-free protein block (Dako, Glostrup, Denmark) at room temperature (RT) for 10 min and then incubated at 4°C overnight with a rabbit anti-RKIP (1:250, Abcam) and with a rabbit anti-pRKIP (1:100, LSBio). Binding of the secondary biotinylated antibody was detected by the Dako Real EnVision Detection System, Peroxidase/DAB kit (Dako), according to the manufacturer's instructions. Sections were counterstained with Mayer's haematoxylin (blue) and mounted with glycerol (Dako Cytomation). Negative controls were obtained by incubating serial sections with the blocking solution and then omitting the primary antibodies. Staining of histological sections was evaluated by optical light microscope using a Leica microscope fitted with a Coolpix 990 digital camera (Nikon). Protein immunoreactivity was initially scored on the extent and intensity of staining, which was graded on an arbitrary scale ranging from 0 to 3, with 0 = negative, 1 = low, 2 = medium, and 3 = high expression. Image processing and immunohistochemical staining quantification were performed with Adobe® Photoshop® CS4. The percent of stained area to total area was calculated and the values of all consecutive images of each core were averaged.

### Statistical analysis

Statistical calculations were performed with MedCalc 9.2.0.1 (MedCalc software, Mariakerke, Belgium) and PASW 18 software (PASW 18, SPSS, Chicago, Ill, USA). Comparisons of RKIP median values between different groups were evaluated by Mann–Whitney U test. Receiver Operating Characteristic (ROC) curve analysis was performed to identify the RKIP cut-off for survival stratification. In the cancer-specific survival (CSS) analysis, patients still alive or lost to follow-up were censored, as well as patients who died of ccRCC-unrelated causes. Progression-free survival (PFS) was calculated from the date of surgery to the date of disease recurrence. Estimates of CSS and PFS were calculated according to the Kaplan-Meier method and compared with the log-rank test. Spearman's correlation was applied to evaluate associations between RKIP and tumor size/grade. Univariate and multivariate analyses were performed using the Cox proportional hazards regression model to identify the most significant variables for predicting CSS and PFS. The backward selection procedure with removal criterion *P* > 0.10 based on likelihood ratio tests was performed. A *P*-value < 0.05 was considered statistically significant.

The present work has been financed by the project “Strategie innovative ad alta tecnologia per lo studio del carcinoma renale. Uso degli OMICS e della biologia dei sistemi per lo sviluppo di nuovi biomarkers” granted to E.R. (code RBAP11B2SX ) of the Italian Ministry of University and Research (MIUR).

## SUPPLEMENTARY MATERIALS FIGURES AND TABLES





## References

[1] Siegel RL, Miller KD, Jemal A (2016). Cancer statistics, 2016. CA Cancer J Clin.

[2] Hunt JD, van der Hel OL, McMillan GP, Boffetta P, Brennan P (2005). Renal cell carcinoma in relation to cigarette smoking: meta-analysis of 24 studies. Int J Cancer.

[3] Renehan AG, Tyson M, Egger M, Heller RF, Zwahlen M (2008). Body-mass index and incidence of cancer: a systematic review and meta-analysis of prospective observational studies. Lancet.

[4] Weikert S, Boeing H, Pischon T, Weikert C, Olsen A, Tjonneland A, Overvad K, Becker N, Linseisen J, Trichopoulou A, Mountokalakis T, Trichopoulos D, Sieri S (2008). Blood pressure and risk of renal cell carcinoma in the European prospective investigation into cancer and nutrition. Am J Epidemiol.

[5] Vavallo A, Simone S, Lucarelli G, Rutigliano M, Galleggiante V, Grandaliano G, Gesualdo L, Campagna M, Cariello M, Ranieri E, Pertosa G, Lastilla G, Selvaggi FP (2014). Pre-existing type 2 diabetes mellitus is an independent risk factor for mortality and progression in patients with renal cell carcinoma. Medicine (Baltimore).

[6] Breda A, Lucarelli G, Rodriguez-Faba O, Guirado L, Facundo C, Bettocchi C, Gesualdo L, Castellano G, Grandaliano G, Battaglia M, Palou J, Ditonno P, Villavicencio H (2015). Clinical and pathological outcomes of renal cell carcinoma (RCC) in native kidneys of patients with end stage renal disease: a long-term comparative retrospective study with RCC diagnosed in the general population. World J Urol.

[7] Weiss RH, Lin PY (2006). Kidney cancer: identification of novel targets for therapy. Kidney Int.

[8] Flanigan RC, Campbell SC, Clark JI, Picken MM (2003). Metastatic renal cell carcinoma. Curr Treat Options Oncol.

[9] Battaglia M, Lucarelli G (2015). The role of renal surgery in the era of targeted therapy: the urologist's perspective. Urologia.

[10] Zhou L, Cai X, Liu Q, Jian ZY, Li H, Wang KJ (2015). Prognostic Role of C-Reactive Protein In Urological Cancers: A Meta-Analysis. Sci Rep.

[11] Lucarelli G, Ditonno P, Bettocchi C, Vavallo A, Rutigliano M, Galleggiante V, Larocca AM, Castellano G, Gesualdo L, Grandaliano G, Selvaggi FP, Battaglia M (2014). Diagnostic and prognostic role of preoperative circulating CA 15-3, CA 125, and beta-2 microglobulin in renal cell carcinoma. Dis Markers.

[12] Gigante M, Lucarelli G, Divella C, Netti GS, Pontrelli P, Cafiero C, Grandaliano G, Castellano G, Rutigliano M, Stallone G, Bettocchi C, Ditonno P, Gesualdo L (2015). Soluble Serum αKlotho Is a Potential Predictive Marker of Disease Progression in Clear Cell Renal Cell Carcinoma. Medicine (Baltimore).

[13] Lucarelli G, Galleggiante V, Rutigliano M, Sanguedolce F, Cagiano S, Bufo P, Lastilla G, Maiorano E, Ribatti D, Giglio A, Serino G, Vavallo A, Bettocchi C (2015). Metabolomic profile of glycolysis and the pentose phosphate pathway identifies the central role of glucose-6-phosphate dehydrogenase in clear cell-renal cell carcinoma. Oncotarget.

[14] Lucarelli G, Rutigliano M, Sanguedolce F, Galleggiante V, Giglio A, Cagiano S, Bufo P, Maiorano E, Ribatti D, Ranieri E, Gigante M, Gesualdo L, Ferro M (2015). Increased Expression of the Autocrine Motility Factor is Associated With Poor Prognosis in Patients With Clear Cell-Renal Cell Carcinoma. Medicine (Baltimore).

[15] Su Kim D, Choi YD, Moon M, Kang S, Lim JB, Kim KM, Park KM, Cho NH (2013). Composite three-marker assay for early detection of kidney cancer. Cancer Epidemiol Biomarkers Prev.

[16] Minamida S, Iwamura M, Kodera Y, Kawashima Y, Tabata K, Matsumoto K, Fujita T, Satoh T, Maeda T, Baba S (2011). 14-3-3 protein beta/alpha as a urinary biomarker for renal cell carcinoma: proteomic analysis of cyst fluid. Anal Bioanal Chem.

[17] Gianazza E, Chinello C, Mainini V, Cazzaniga M, Squeo V, Albo G, Signorini S, Di Pierro SS, Ferrero S, Nicolardi S, van der Burgt YE, Deelder AM, Magni F (2012). Alterations of the serum peptidome in renal cell carcinoma discriminating benign and malignant kidney tumors. J Proteomics.

[18] Teng PN, Hood BL, Sun M, Dhir R, Conrads TP (2011). Differential proteomic analysis of renal cell carcinoma tissue interstitial fluid. J Proteome Res.

[19] Okamura N, Masuda T, Gotoh A, Shirakawa T, Terao S, Kaneko N, Suganuma K, Watanabe M, Matsubara T, Seto R, Matsumoto J, Kawakami M, Yamamori M (2008). Quantitative proteomic analysis to discover potential diagnostic markers and therapeutic targets in human renal cell carcinoma. Proteomics.

[20] Papale M, Pedicillo MC, Thatcher BJ, Di Paolo S, Lo Muzio L, Bufo P, Rocchetti MT, Centra M, Ranieri E, Gesualdo L (2007). Urine profiling by SELDI-TOF/MS: monitoring of the critical steps in sample collection, handling and analysis. J Chromatogr B Analyt Technol Biomed Life Sci.

[21] Papale M, Rocchetti MT, Gesualdo L (2013). Clinical proteomics: the potentiality of urine analysis for understanding diabetic nephropathy. EMJ Nephr.

[22] Rocchetti MT, Tamma G, Lasorsa D, Suriano IV, D’Apollo A, Papale M, Mastrofrancesco L, Grandaliano G, Svelto M, Valenti G, Gesualdo L, Di Paolo S (2011). Altered urinary excretion of aquaporin 2 in IgA nephropathy. Eur J Endocrinol.

[23] Papale M, Di Paolo S, Magistroni R, Lamacchia O, Di Palma AM, De Mattia A, Rocchetti MT, Furci L, Pasquali S, De Cosmo S, Cignarelli M, Gesualdo L (2010). Urine proteome analysis may allow noninvasive differential diagnosis of diabetic nephropathy. Diabetes Care.

[24] Chinello C, Cazzaniga M, De Sio G, Smith AJ, Gianazza E, Grasso A, Rocco F, Signorini S, Grasso M, Bosari S, Zoppis I, Dakna M, van der Burgt YE (2014). Urinary signatures of Renal Cell Carcinoma investigated by peptidomic approaches. PLoS One.

[25] Deiss K, Kisker C, Lohse MJ, Lorenz K (2012). Raf kinase inhibitor protein (RKIP) dimer formation controls its target switch from Raf1 to G protein-coupled receptor kinase (GRK) 2. J Biol Chem.

[26] Hill B, De Melo J, Yan J, Kapoor A, He L, Cutz JC, Feng X, Bakhtyar N, Tang D (2014). Common reduction of the Raf kinase inhibitory protein in clear cell renal cell carcinoma. Oncotarget.

[27] Kriz W, Kaissling B, Le Hir M (2011). Epithelial-mesenchymal transition (EMT) in kidney fibrosis: fact or fantasy?. J Clin Invest.

[28] Ho MY, Tang SJ, Chuang MJ, Cha TL, Li JY, Sun GH, Sun KH (2012). TNF-α induces epithelial-mesenchymal transition of renal cell carcinoma cells via a GSK3β-dependent mechanism. Mol Cancer Res.

[29] Wu K, Bonavida B (2009). The activated NF-kappaB-Snail-RKIP circuitry in cancer regulates both the metastatic cascade and resistance to apoptosis by cytotoxic drugs. Crit Rev Immunol.

[30] Lee HC, Tian B, Sedivy JM, Wands JR, Kim M (2006). Loss of Raf kinase inhibitor protein promotes cell proliferation and migration of human hepatoma cells. Gastroenterology.

[31] Lamouille S, Xu J, Derynck R (2014). Molecular mechanisms of epithelial-mesenchymal transition. Nat Rev Mol Cell Biol.

[32] Penela P, Murga C, Ribas C, Lafarga V, Mayor F (2010). The complex G protein-coupled receptor kinase 2 (GRK2) interactome unveils new physiopathological targets. Br J Pharmacol.

[33] Rocchetti MT, Centra M, Papale M, Bortone G, Palermo C, Centonze D, Ranieri E, Di Paolo S, Gesualdo L (2008). Urine protein profile of IgA nephropathy patients may predict the response to ACE-inhibitor therapy. Proteomics.

[34] Shevchenko A, Wilm M, Vorm O, Mann M (1996). Mass spectrometric sequencing of proteins silver-stained polyacrylamide gels. Anal Chem.

