# Supplementary material for: Urinary RKIP/p-RKIP is a potential diagnostic and prognostic marker of clear cell renal cell carcinoma
Source: Oncotarget. 2017 Mar 18;8(25):40412–24. doi: 10.18632/oncotarget.16341 (PMC5522321; doi:10.18632/oncotarget.16341)
Supplement: Supplementary file 1 [file oncotarget-08-40412-s001.pdf]

Urinary RKIP/p-RKIP is a potential diagnostic and prognostic marker of clear cell renal cell carcinoma

SUPPLEMENTARY MATERIALS

File: **whole set.csv**
Target Variable: **C0GRP_NA$**
Predictor Variables: **C08956_9, C023322_**

A)

Tree Sequence

Tree Number	Terminal Nodes	Cross-Validated Relative Cost	Resubstitution Relative Cost	Complexity
1	4	0.348 ± 0.103	0.139	-1.000
2**	3	0.321 ± 0.100	0.146	0.004
3	2	0.422 ± 0.111	0.258	0.056
4	1	1.000 ± 0.000	1.000	0.371

*** Minimum Cost**
**** Optimal**

Data Sample

Class	Learn	%	Total
HS	36	62.069	36
ccRCC	22	37.931	22
Total:	58	100.000	58

B)

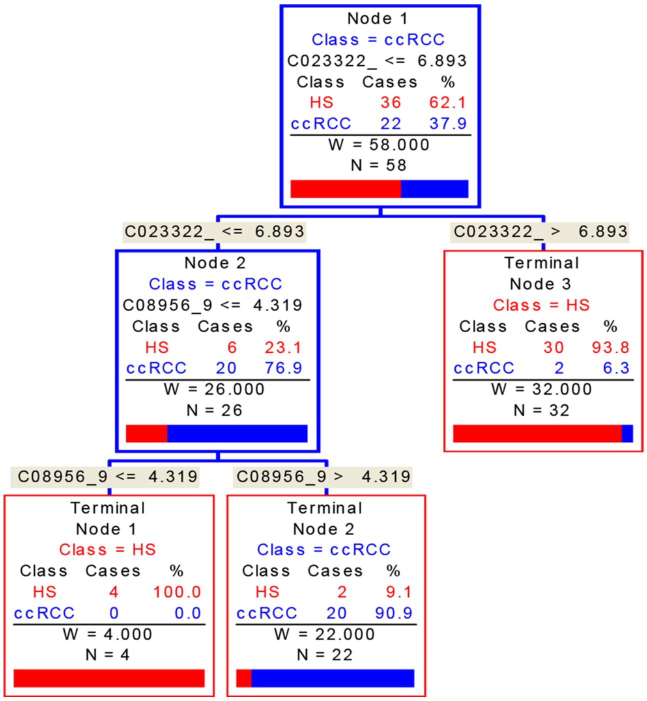

(Continued)

C)

Prediction Success

**Actual Class**	**Total Cases**	**Percent Correct**	**HS N=36**	**ccRCC N=22**
**HS**	**36**	**94.444**	**34**	**2**
**ccRCC**	**22**	**90.909**	**2**	**20**

D)

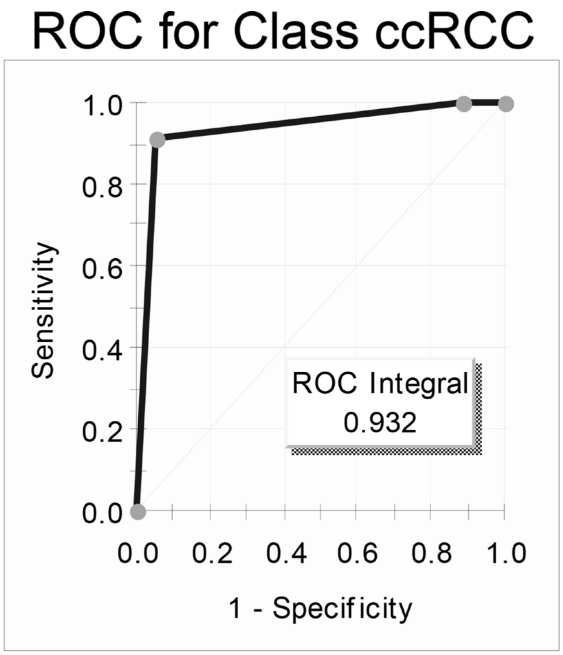

File: training set 1.csv
Target Variable: C0GRP_NA$
Predictor Variables: C08956_9, C023322_

E)

Tree Sequence

**Tree Number**	**Terminal Nodes**	**Test Set Relative Cost**	**Resubstitution Relative Cost**	**Complexity**
1	4	0.196 ± 0.136	0.136	-1.000
2**	3	0.125 ± 0.117	0.162	0.013
3	2	0.268 ± 0.150	0.253	0.045
4	1	1.000 ± 0.000	1.000	0.373

* Minimum Cost
** Optimal

(*Continued*)

Data Sample

Class	Learn	%	Test	%	Total
HS	22	61.111	14	63.636	36
ccRCC	14	38.889	8	36.364	22
Total:	36	100.000	22	100.000	58

F)

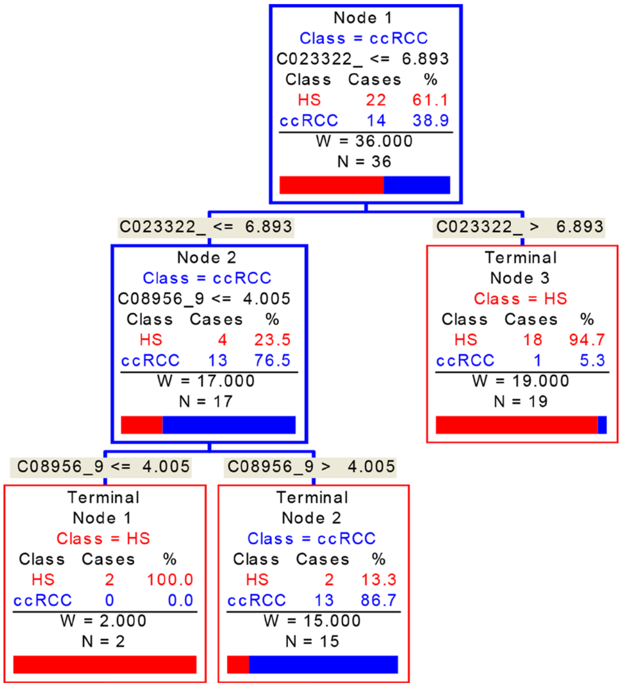

G)

Prediction Success (training 1)

Actual Class	Total Cases	Percent Correct	HS N=21	ccRCC N=15
HS	22	90.909	20	2
ccRCC	14	92.857	1	13

Prediction Success (testing 1)

Actual Class	Total Cases	Percent Correct	HS N=15	ccRCC N=7
HS	14	100.000	14	0
ccRCC	8	87.500	1	7

(*Continued*)

H)

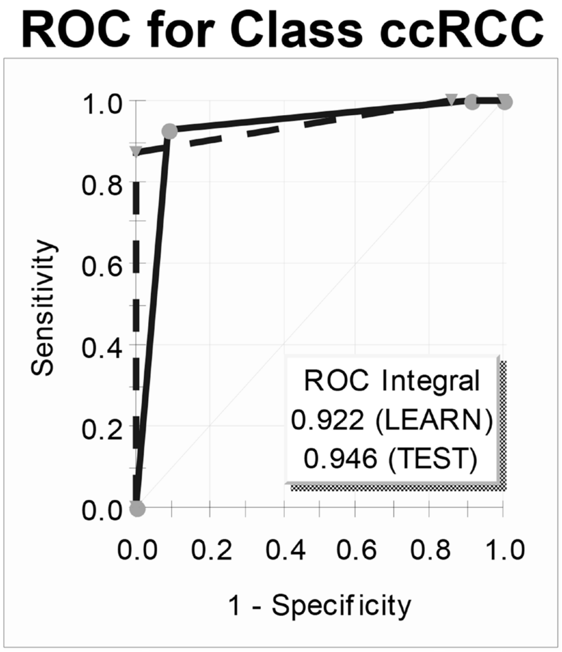

File: training set 2.csv
Target Variable: C0GRP_NA$
Predictor Variables: C08956_9, C023322_

I)

Tree Sequence

Tree Number	Terminal Nodes	Test Set Relative Cost	Resubstitution Relative Cost	Complexity
1	4	0.411 ± 0.168	0.000	-1.000
2**	3	0.268 ± 0.150	0.071	0.036
3	2	0.339 ± 0.160	0.208	0.068
4	1	1.000 ± 0.000	1.000	0.396

* Minimum Cost
** Optimal

Data Sample

Class	Learn	%	Test	%	Total
HS	22	61.111	14	63.636	36
ccRCC	14	38.889	8	36.364	22
Total:	36	100.000	22	100.000	58

(*Continued*)

J)

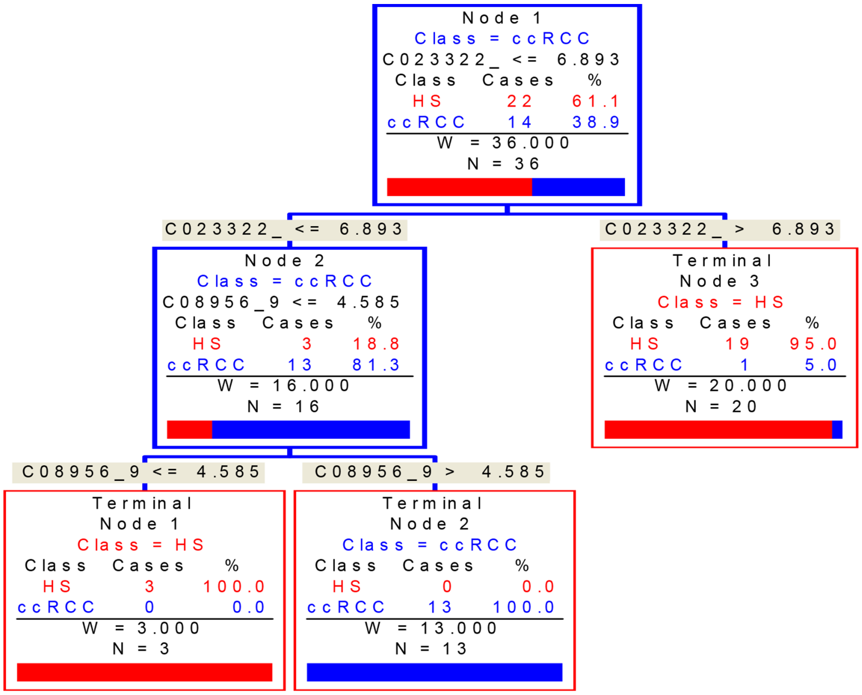

K)

Prediction Success (training 2)

Actual Class	Total Cases	Percent Correct	HS N=23	ccRCC N=13
HS	22	100.000	22	0
ccRCC	14	92.857	1	13

Prediction Success (testing 2)

Actual Class	Total Cases	Percent Correct	HS N=13	ccRCC N=9
HS	14	85.714	12	2
ccRCC	8	87.500	1	7

(*Continued*)

L)

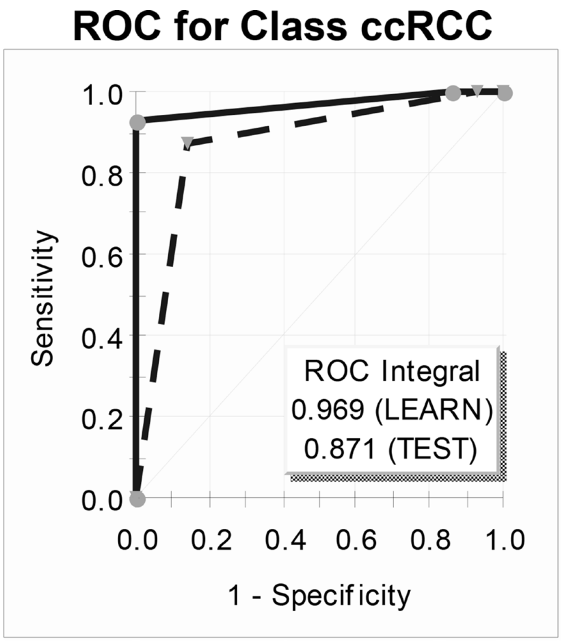

File: training set 3.csv
Target Variable: C0GRP_NA$
Predictor Variables: C08956_9, C023322_

M)

Tree Sequence

Tree Number	Terminal Nodes	Test Set Relative Cost	Resubstitution Relative Cost	Complexity
1	4	0.196 ± 0.136	0.136	-1.000
2**	3	0.125 ± 0.117	0.188	0.026
3	2	0.196 ± 0.136	0.325	0.068
4	1	1.000 ± 0.000	1.000	0.338

* Minimum Cost
** Optimal

Data Sample

Class	Learn	%	Test	%	Total
HS	22	61.111	14	63.636	36
ccRCC	14	38.889	8	36.364	22
Total:	36	100.000	22	100.000	58

(*Continued*)

N)

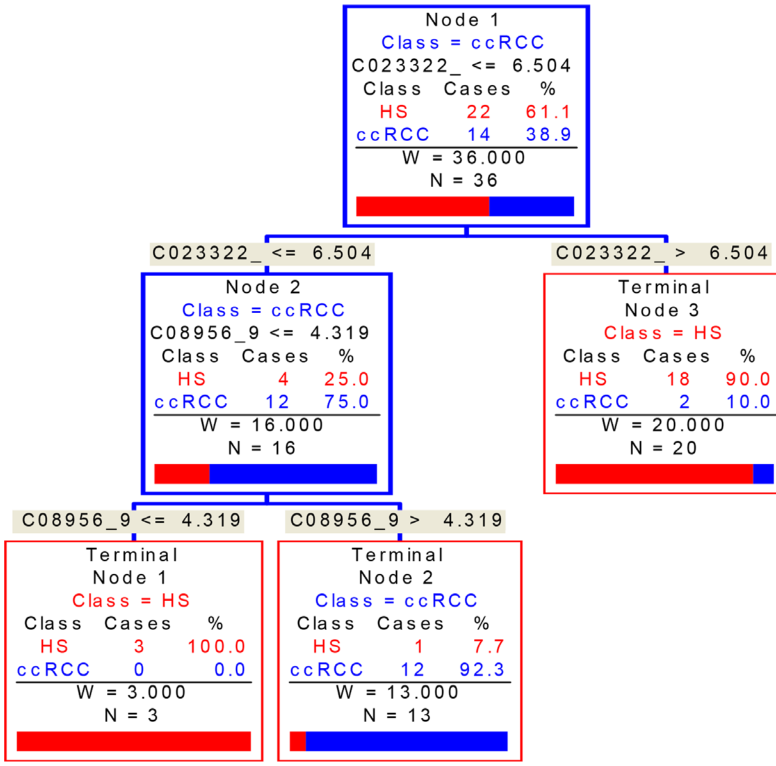

O)

Prediction Success (training 3)

Actual Class	Total Cases	Percent Correct	HS N=23	ccRCC N=13
HS	22	95.455	21	1
ccRCC	14	85.714	2	12

Prediction Success (testing 3)

Actual Class	Total Cases	Percent Correct	HS N=15	ccRCC N=7
HS	14	100.000	14	0
ccRCC	8	87.500	1	7

(Continued)

P)

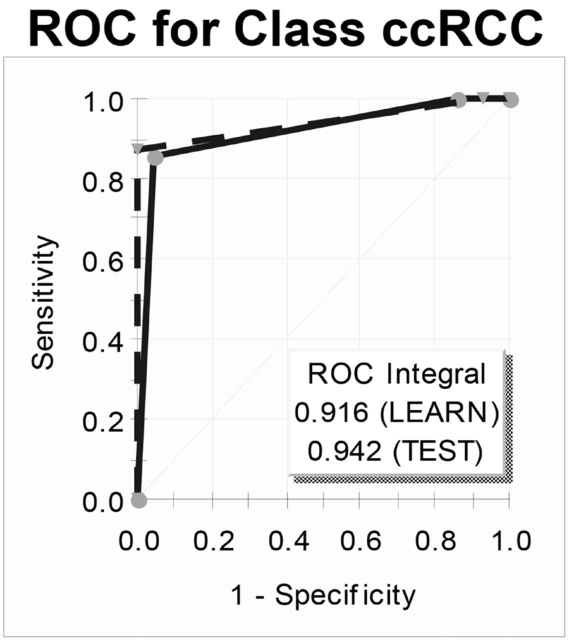

Supplementary Figure 1: Classification and Regression Tree analysis (CART). CART analysis carried out on 22 ccRCC Vs. 36 HS samples and its performances after 10 times cross-validation is reported in panels **(A-D).** Furthermore, the samples were randomly assigned to a training (22 HS vs. 14 ccRCC) and a testing set (14 HS Vs. 8 ccRCC) and run, independently, for three times. Tables **(E)**, **(I)**, **(M)** report the characteristics of the trees and the number of HS and ccRCC used in the training and testing set, respectively. The graphic representation of the tree used for each analysis is reported in panels **(F)**, **(J)** and **(N)**, respectively. Panels **(G)**, **(K)**, and **(O)** show the prediction success observed in each case. Finally, The ROC curves of each analysis are shown in panels **(H)**, **(L)** and **(P)**, respectively. The power of the test for the prediction of HS and ccRCC ranges from 87% **(panel (L))** to 94% **(panels (H)** and **(P))**.

A

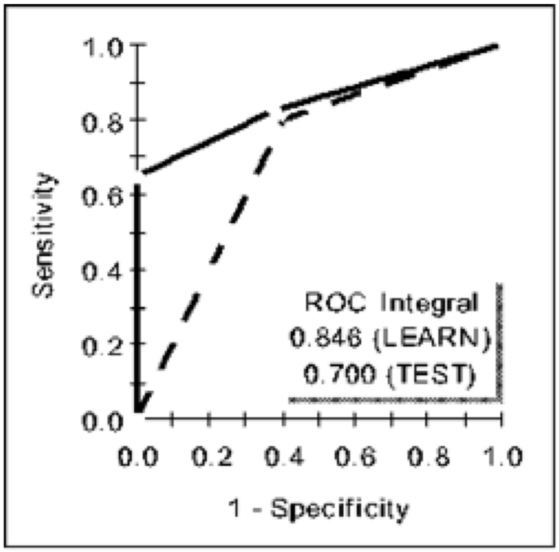

B

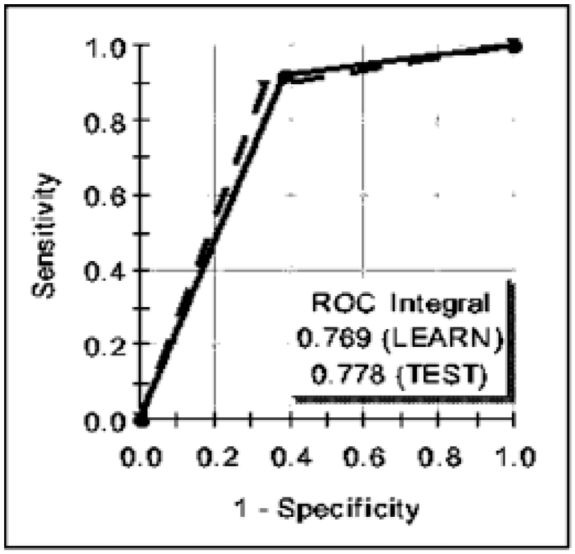

C

Training set

Actual Class	Total Cases	Percent Correct	PCa N=14	ccRCC N=11
PCa	8	100.000	8	0
ccRCC	17	64.706	6	11

D

Testing set (internal cross-validation)

Actual Class	Total Cases	Percent Correct	PCa N=7	ccRCC N=3
PCa	5	80.000	4	1
ccRCC	5	40.000	3	2

E

Training set

Actual Class	Total Cases	Percent Correct	CKD N=9	ccRCC N=17
CKD	13	61.538	8	5
ccRCC	13	92.308	1	12

F

Testing set

Actual Class	Total Cases	Percent Correct	CKD N=7	ccRCC N=11
CKD	9	66.667	6	3
ccRCC	9	88.889	1	8

G

Variable importance

Variable	Score	
C023322_	100.00	
C08956_9	20.40	

H

Variable importance

Variable	Score	
C023322_	100.00	
C08956_9	5.92	

Supplementary Figure 2: CART analysis in ccRCC *Vs* PCa and CKD. ROC curve obtained by CART analysis in ccRCC vs Prostate cancer (PCa) **(A)** and ccRCC vs chronic kidney disease (CKD) **(B)**. Results of CART analysis carried out by 10 times internal cross-validation on 20% of randomly extracted samples (ccRCC vs PCa) or by applying the classification tree on an independent testing set (ccRCC Vs CKD) are reported in **panels (C-F)**. The mass peak of 23320 m/z (RKIP) was the most important variable in both the analyses **(panels (G-H))**.

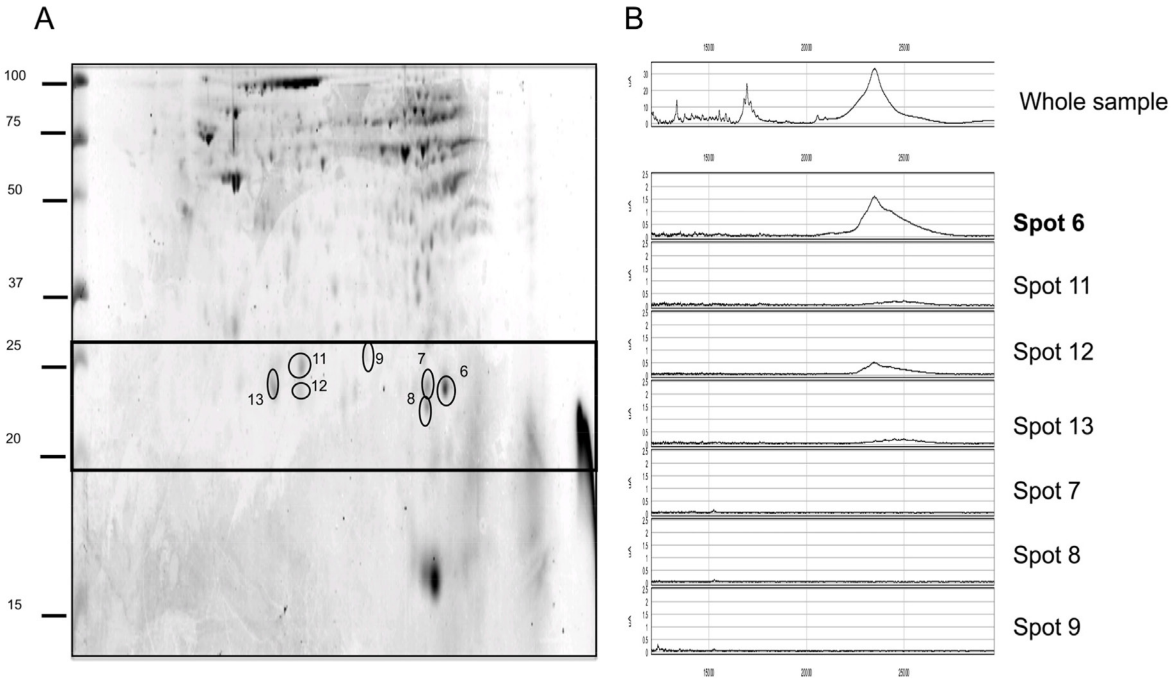

Supplementary Figure 3: Purification of the 23,300 m/z mass peak. (A) Two dimensional gel map of the urine samples in one healthy subject. Gel spots in the mass range between 20 and 25 kDa are indicated in the rectangle. The gel spots cut and eluted from the gel by passive elution are shown in the circles. **(B)** Upper panel: SELDI protein profile of the healthy subject obtained with the CM10 ProteinChip Array. Lower panels: CM10 protein profile of each spot eluted from the gel. The gel spot identified by MALDI-TOF/MS/MS analysis as RKIP is indicated in bold.

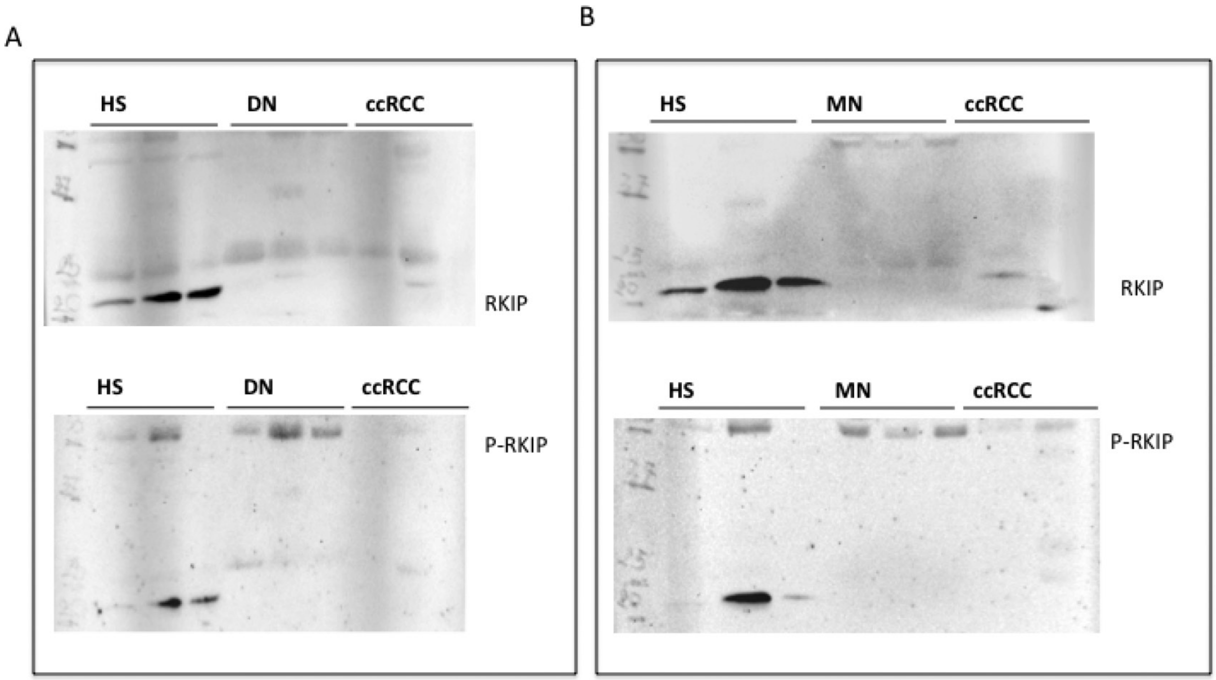

Supplementaty Figure 4: Full size western blotting. Full-size western blotting on RKIP and p-RKIP in ccRCC vs DN **(panel A)** and ccRCC vs MN **(panel B)**.

Supplementary Table 1: List of mass peaks shared (clusters) between ccRCC and Healthy Subjects (HS)

The FDR was calculated according to Benjamini & Hochberg's method. The two predictors used to build the classification and regression tree (CART) are marked in bold.

See Supplementary File 1

Supplementary Table 2: Sensitivity and specificity of RKIP for cancer-specific survival (CSS) and progression-free survival (PFS)

	Sensitivity	95% CI	Specificity	95% CI
**CSS**	75%	42.8-94.2	86%	72.1-94.7
**PFS**	68.7%	41.4-88.9	84.6 %	69.5-94.1

Supplementary Table 3: Univariate and multivariate analyses for cancer-specific survival

Characteristic	Univariate		Multivariate	
	HR (95% CI)	P-value	HR (95% CI)	P-value
**Age at surgery (<65 vs ≥65 years)**	1.22 (1.06-2.75)	0.02		0.69
**Gender (male vs female)**	0.62 (0.32-1.19)	0.28		NA
**Tumor size (< 7 vs ≥7 cm)**	3.51 (2.37-8.41)	<0.001	1.63 (1.02-4.43)	<0.001
**pT stage (T1-2 vs T3-4)**	4.33 (2.14-11.21)	<0.001		0.56
**Distant metastasis (M0 vs M1)**	10.28 (4.92-18.34)	<0.001	5.28 (2.18-10.32)	<0.001
**TNM stage (I-II vs III-IV)**	5.35 (2.25-9.74)	<0.001		0.36
**Fuhrman grade (1-2 vs 3-4)**	4.32 (2.65-9.18)	<0.001	3.26 (1.85-6.49)	0.01
**RKIP expression (High vs low)**	2.50 (1.34-5.22)	<0.001	1.23 (1.03-3.62)	0.02

Supplementary Table 4: Univariate and multivariate analyses for progression-free survival

Characteristic	Univariate		Multivariate	
	HR (95% CI)	P-value	HR (95% CI)	P-value
Age at surgery (<65 vs ≥65 years)	1.12 (0.66-3.29)	0.68		NA
Gender (male vs female)	0.68 (0.41-1.27)	0.32		NA
Tumor size (< 7 vs ≥7 cm)	4.26 (2.21-9.45)	<0.001	3.29 (1.88-7.49)	<0.001
pT stage (T1-2 vs T3-4)	8.93 (4.34-18.38)	<0.001		0.32
Distant metastasis (M0 vs M1)	12.29 (6.22-23.54)	<0.001	6.22 (2.12-13.28)	<0.001
TNM stage (I-II vs III-IV)	10.32 (4.37-23.14)	<0.001		0.37
Fuhrman grade (1-2 vs 3-4)	3.11 (1.64-6.62)	<0.001		0.64
RKIP expression (High vs low)	2.25 (1.36-6.27)	<0.001	1.38 (1.05-4.21)	0.02

Supplementaty Table 5: Clinical and pathological characteristics of the patients enrolled for the discovery phase of study

	ccRCC	PCa	CKD	HS
**Patients, n**	22	13	22	36
**Age (years)**				
**median**	63.5	66	57	31
**95% CI**	60.2-71	64.4-71.2	48.6-63	29-35
**Gender**				
**Male**	15 (68%)	13 (100%)	14 (64%)	17 (47%)
**Female**	7 (32%)	/	8 (36%)	19 (53%)
**Creatinine (Urine), mg/dL**				
**median**	106	158.8	60.7	144.2
**95%CI**	78-151.4	73.8-190.8	41.5-81.8	120.6-185.6
**Protein (Urine), mg/dL**				
**median**	4.9	8.5	56	9.6
**95%CI**	3.1-9.8	1.7-15.7	40.2-229.5	6.0-10.1
**TNM stage**				
**I**	2 (9%)	/		
**II**	11 (50%)	8 (61%)	/	/
**III**	8 (36%)	5 (39%)		
**IV**	1 (5%)	/		
**Gleason score, n (7/8/9)**	/	8/3/2	/	/
**Fuhrman grade**				
**G1-2**	12 (54.5%)	/	/	/
**G3-4**	10 (35.5%)			
**Total PSA, ng/mL**				
**median**	/	12.4	/	/
**95%CI**				

Supplementary Table 6: Clinical and pathological characteristics of ccRCC patients enrolled for the validation phase of the study

Patients	n=56
Age (years)	
median	57
95% CI	46.6 - 64
Gender	
Male	37 (66%)
Female	19 (34%)
Tumor size (cm)	
median	5.2
95% CI	4.5 - 6.7
TNM stage	
I	29 (52%)
II	9 (16%)
III	7 (12.5%)
IV	11 (19.5%)
pN+	11 (19.5%)
cM+	10 (17.8%)
Fuhrman grade	
G1-2	36 (64.3%)
G3-4	20 (35.7%)
Follow-up	
Median (months)	41
95% CI	29.9 – 45.0
