# Supplementary material for: Urinary RKIP/p-RKIP is a potential diagnostic and prognostic marker of clear cell renal cell carcinoma
Source: Oncotarget. 2017 Mar 18;8(25):40412–24. doi: 10.18632/oncotarget.16341 (PMC5522321; doi:10.18632/oncotarget.16341)
Supplement: Supplementary file 2 [file oncotarget-08-40412-s002.docx]

**Table S1**

| **Peak n.** | **Peak ID** | **Peak (m/z)** | **p- value** | **fdr (BH)** | **ROC** | **Trend in ccRCC** | **Fold-change** |
| --- | --- | --- | --- | --- | --- | --- | --- |
| **1** | **99** | **23322** | **1,17E-07** | **9,60E-06** | **0,9** | **Decreased** | **-4.1** |
| 2 | 29 | 5570 | 1,95E-07 | 9,60E-06 | 0,1 | Increased | 7 |
| 3 | 98 | 22768 | 3,22E-07 | 9,60E-06 | 0,9 | Decreased | -2.6 |
| 4 | 27 | 5371 | 3,80E-07 | 9,60E-06 | 0,1 | Increased | 4.6 |
| 5 | 13 | 4128 | 6,21E-07 | 1,25E-05 | 0,1 | Increased | 4 |
| 6 | 45 | 6873 | 9,28E-07 | 1,56E-05 | 0,1 | Increased | 4.8 |
| 7 | 30 | 5701 | 1,09E-06 | 1,57E-05 | 0,1 | Increased | 3.9 |
| 8 | 47 | 7050 | 1,49E-06 | 1,81E-05 | 0,1 | Increased | 5.3 |
| 9 | 7 | 3737 | 1,61E-06 | 1,81E-05 | 0,1 | Increased | 3.5 |
| **10** | **69** | **8957** | **4,37E-06** | **4,41E-05** | **0,2** | **Increased** | **3.5** |
| 11 | 66 | 8755 | 5,90E-06 | 5,42E-05 | 0,2 | Increased | 2.7 |
| 12 | 65 | 8567 | 6,84E-06 | 5,76E-05 | 0,2 | Increased | 2.3 |
| 13 | 49 | 7311 | 1,14E-05 | 8,80E-05 | 0,2 | Increased | 3.5 |
| 14 | 52 | 7485 | 1,22E-05 | 8,80E-05 | 0,1 | Increased | 4.1 |
| 15 | 12 | 4031 | 1,31E-05 | 8,82E-05 | 0,2 | Increased | 3.2 |
| 16 | 2 | 3080 | 1,52E-05 | 9,60E-05 | 0,2 | Increased | 5.1 |
| 17 | 34 | 6163 | 4,01E-05 | 0,000238241 | 0,8 | Decreased | -2.3 |
| 18 | 6 | 3590 | 6,00E-05 | 0,000318947 | 0,2 | Increased | 2.6 |
| 19 | 25 | 5233 | 6,00E-05 | 0,000318947 | 0,2 | Increased | 6.1 |
| 20 | 16 | 4355 | 7,32E-05 | 0,000366235 | 0,2 | Increased | 3.2 |
| 21 | 5 | 3474 | 8,34E-05 | 0,000366235 | 0,2 | Increased | 3.1 |
| 22 | 79 | 10654 | 8,34E-05 | 0,000366235 | 0,8 | Decreased | -3.7 |
| 23 | 81 | 10845 | 8,34E-05 | 0,000366235 | 0,8 | Decreased | -2 |
| 24 | 92 | 15052 | 0,000179 | 0,0007272 | 0,8 | Decreased | -2.4 |
| 25 | 8 | 3820 | 0,000191 | 0,0007272 | 0,2 | Increased | 2.1 |
| 26 | 24 | 5082 | 0,000203 | 0,0007272 | 0,2 | Increased | 4.8 |
| 27 | 70 | 9062 | 0,000203 | 0,0007272 | 0,2 | Increased | 2.4 |
| 28 | 3 | 3346 | 0,000216 | 0,0007272 | 0,2 | Increased | 3 |
| 29 | 9 | 3890 | 0,000216 | 0,0007272 | 0,2 | Increased | 3.1 |
| 30 | 53 | 7560 | 0,000216 | 0,0007272 | 0,2 | Increased | 2.8 |
| 31 | 1 | 3028 | 0,000245 | 0,000798226 | 0,2 | Increased | 9 |
| 32 | 32 | 5952 | 0,000312 | 0,000954909 | 0,2 | Increased | 1.8 |
| 33 | 72 | 9229 | 0,000312 | 0,000954909 | 0,2 | Increased | 3.2 |
| 34 | 14 | 4173 | 0,000352 | 0,000987556 | 0,2 | Increased | 3.6 |
| 35 | 43 | 6712 | 0,000352 | 0,000987556 | 0,2 | Increased | 1.9 |
| 36 | 44 | 6823 | 0,000352 | 0,000987556 | 0,2 | Increased | 2.9 |
| 37 | 67 | 8843 | 0,000473 | 0,001291162 | 0,3 | Increased | 3.1 |
| 38 | 59 | 8020 | 0,000532 | 0,001414 | 0,8 | Decreased | -1.5 |
| 39 | 15 | 4305 | 0,00105274 | 0,002726327 | 0,3 | Increased | 2.7 |
| 40 | 48 | 7076 | 0,001462668 | 0,003693237 | 0,3 | Increased | 2.4 |
| 41 | 84 | 11376 | 0,002124401 | 0,005233281 | 0,7 | Decreased | -2.2 |
| 42 | 4 | 3439 | 0,002358591 | 0,00567185 | 0,3 | Increased | 2.6 |
| 43 | 90 | 13388 | 0,002484344 | 0,00583532 | 0,7 | Decreased | -1.8 |
| 44 | 85 | 11484 | 0,002616204 | 0,006005377 | 0,7 | Decreased | -4.7 |
| 45 | 39 | 6518 | 0,002754433 | 0,006182172 | 0,7 | Decreased | -5.2 |
| 46 | 54 | 7660 | 0,003051097 | 0,006699148 | 0,3 | Increased | 2.7 |
| 47 | 68 | 8865 | 0,003733494 | 0,00802304 | 0,3 | Increased | 1.8 |
| 48 | 64 | 8456 | 0,004780425 | 0,010058811 | 0,3 | Increased | 1.6 |
| 49 | 31 | 5813 | 0,005268861 | 0,010860305 | 0,3 | Increased | 1.6 |
| 50 | 93 | 15873 | 0,00580194 | 0,011719919 | 0,7 | Decreased | -1.7 |
| 51 | 46 | 6973 | 0,006383172 | 0,012641184 | 0,3 | Increased | 4.4 |
| 52 | 63 | 8384 | 0,009699933 | 0,018840254 | 0,3 | Increased | 1.6 |
| 53 | 82 | 10970 | 0,010150147 | 0,019342733 | 0,7 | Decreased | -1.9 |
| 54 | 11 | 3991 | 0,01110675 | 0,020773736 | 0,3 | Increased | 4.5 |
| 55 | 37 | 6331 | 0,012142612 | 0,021900068 | 0,3 | Increased | 1.4 |
| 56 | 95 | 16945 | 0,012142612 | 0,021900068 | 0,7 | Decreased | -2.1 |
| 57 | 35 | 6179 | 0,013263197 | 0,023096257 | 0,7 | Decreased | -2.3 |
| 58 | 83 | 11036 | 0,013263197 | 0,023096257 | 0,7 | Decreased | -1.6 |
| 59 | 94 | 16806 | 0,014474246 | 0,024777947 | 0,7 | Decreased | -2.4 |
| 60 | 57 | 7916 | 0,015781774 | 0,026565986 | 0,3 | Increased | 1.8 |
| 61 | 36 | 6206 | 0,016473676 | 0,027276086 | 0,7 | Decreased | -1.4 |
| 62 | 80 | 10782 | 0,017192082 | 0,028006456 | 0,7 | Decreased | -1.9 |
| 63 | 86 | 11777 | 0,022106971 | 0,034887564 | 0,7 | Decreased | -1.7 |
| 64 | 96 | 17099 | 0,022106971 | 0,034887564 | 0,7 | Decreased | -2.2 |
| 65 | 56 | 7901 | 0,023997125 | 0,03728784 | 0,3 | Increased | 1.7 |
| 66 | 51 | 7462 | 0,033025213 | 0,050538584 | 0,3 | Increased | 1.9 |
| 67 | 40 | 6532 | 0,034336149 | 0,051760463 | 0,7 | Decreased | -2.8 |
| 68 | 78 | 10564 | 0,046513527 | 0,069086268 | 0,6 | Decreased | -1.4 |
| 69 | 18 | 4474 | 0,048264929 | 0,070648664 | 0,3 | n.s | comparable |
| 70 | 26 | 5290 | 0,050071398 | 0,072245874 | 0,3 | n.s | comparable |
| 71 | 91 | 14407 | 0,062138726 | 0,088394526 | 0,6 | n.s | comparable |
| 72 | 71 | 9125 | 0,096606691 | 0,135517719 | 0,4 | n.s | comparable |
| 73 | 33 | 6086 | 0,099793213 | 0,13620425 | 0,4 | n.s | comparable |
| 74 | 75 | 9952 | 0,099793213 | 0,13620425 | 0,6 | n.s | comparable |
| 75 | 19 | 4538 | 0,109857834 | 0,147941883 | 0,4 | n.s | comparable |
| 76 | 23 | 4957 | 0,120708178 | 0,160414816 | 0,4 | n.s | comparable |
| 77 | 55 | 7676 | 0,144906985 | 0,190072799 | 0,4 | n.s | comparable |
| 78 | 89 | 12686 | 0,153750222 | 0,199086826 | 0,4 | n.s | comparable |
| 79 | 77 | 10361 | 0,167776131 | 0,214498598 | 0,6 | n.s | comparable |
| 80 | 17 | 4426 | 0,177647068 | 0,221510542 | 0,4 | n.s | comparable |
| 81 | 50 | 7361 | 0,177647068 | 0,221510542 | 0,4 | n.s | comparable |
| 82 | 42 | 6663 | 0,221467004 | 0,272782529 | 0,4 | n.s | comparable |
| 83 | 88 | 12551 | 0,227446888 | 0,276772719 | 0,4 | n.s | comparable |
| 84 | 62 | 8302 | 0,259071855 | 0,311503064 | 0,4 | n.s | comparable |
| 85 | 41 | 6587 | 0,293619992 | 0,348889638 | 0,4 | n.s | comparable |
| 86 | 100 | 26723 | 0,346966451 | 0,407483855 | 0,6 | n.s | comparable |
| 87 | 20 | 4579 | 0,371608107 | 0,431407113 | 0,5 | n.s | comparable |
| 88 | 87 | 11947 | 0,520439542 | 0,597322656 | 0,5 | n.s | comparable |
| 89 | 28 | 5489 | 0,530663702 | 0,602213864 | 0,4 | n.s | comparable |
| 90 | 76 | 10080 | 0,540988839 | 0,607109697 | 0,4 | n.s | comparable |
| 91 | 73 | 9777 | 0,551413388 | 0,612008266 | 0,5 | n.s | comparable |
| 92 | 58 | 7979 | 0,627024082 | 0,688363394 | 0,5 | n.s | comparable |
| 93 | 21 | 4632 | 0,649418579 | 0,705282543 | 0,5 | n.s | comparable |
| 94 | 22 | 4752 | 0,695153741 | 0,746920509 | 0,5 | n.s | comparable |
| 95 | 74 | 9882 | 0,706771278 | 0,751409464 | 0,5 | n.s | comparable |
| 96 | 60 | 8045 | 0,742026162 | 0,780673358 | 0,5 | n.s | comparable |
| 97 | 10 | 3916 | 0,789875696 | 0,82244789 | 0,5 | n.s | comparable |
| 98 | 97 | 18250 | 0,801970905 | 0,826521035 | 0,5 | n.s | comparable |
| 99 | 61 | 8184 | 0,925070197 | 0,943758484 | 0,5 | n.s | comparable |
| 100 | 38 | 6419 | 0,937530369 | 0,946905673 | 0,5 | n.s | comparable |
| 101 | 101 | 28736 | 0,950005863 | 0,950005863 | 0,5 | n.s | comparable |

**Table S1** List of mass peaks shared (clusters) between ccRCC and Healthy Subjects (HS). The FDR was calculated according to Benjamini & Hochberg's method. The two predictors used to build the classification and regression tree (CART) are marked in bold.
